# Integrated genomic and transcriptomic analysis reveals unique characteristics of hepatic metastases and pro-metastatic role of complement C1q in pancreatic ductal adenocarcinoma

**DOI:** 10.1186/s13059-020-02222-w

**Published:** 2021-01-04

**Authors:** Jianyu Yang, Ping Lin, Minwei Yang, Wei Liu, Xueliang Fu, Dejun Liu, Lingye Tao, Yanmiao Huo, Junfeng Zhang, Rong Hua, Zhigang Zhang, Yixue Li, Liwei Wang, Jing Xue, Hong Li, Yongwei Sun

**Affiliations:** 1 Department of Biliary-Pancreatic Surgery, Ren Ji Hospital, School of Medicine, Shanghai Jiao Tong University, Shanghai, 200127 China; 2 CAS-MPG Partner Institute for Computational Biology, Shanghai Institute of Nutrition and Health, Shanghai Institutes for Biological Sciences, University of Chinese Academy of Sciences, Chinese Academy of Sciences, Shanghai, 200031 China; 3 State Key Laboratory of Oncogenes and Related Genes, Shanghai Cancer Institute, Ren Ji Hospital, School of Medicine, Shanghai Jiao Tong University, Shanghai, 200240 China; 4 School of Life Sciences and Biotechnology, Shanghai Jiao Tong University, Shanghai, 200240 China; 5 Collaborative Innovation Center for Genetics and Development, Fudan University, Shanghai, 200032 China; 6 Shanghai Center for Bioinformation Technology, Shanghai Academy of Science & Technology, Shanghai, 201203 China; 7 Department of Oncology, Ren Ji Hospital, School of Medicine, Shanghai Jiao Tong University, Shanghai, 200127 China; 8 State Key Laboratory of Oncogenes and Related Genes, Renji-Med X Clinical Stem Cell Research Center, Shanghai Cancer Institute, Shanghai Jiao Tong University School of Medicine Affiliated Renji Hospital, Shanghai, 200240 China

**Keywords:** Pancreatic ductal adenocarcinoma, Hepatic metastasis, Genomics, Transcriptomics, Tumor microenvironment, C1q

## Abstract

**Background:**

Pancreatic ductal adenocarcinoma (PDAC) is one of the most lethal cancers due to its high metastasis rate in the liver. However, little is known about the molecular features of hepatic metastases due to difficulty in obtaining fresh tissues and low tumor cellularity.

**Results:**

We conduct exome sequencing and RNA sequencing for synchronous surgically resected primary tumors and the paired hepatic metastases from 17 hepatic oligometastatic pancreatic ductal adenocarcinoma and validate our findings in specimens from 35 of such cases. The comprehensive analysis of somatic mutations, copy number alterations, and gene expressions show high similarity between primary tumors and hepatic metastases. However, hepatic metastases also show unique characteristics, such as a higher degree of 3p21.1 loss, stronger abilities of proliferation, downregulation of epithelial to mesenchymal transition activity, and metabolic rewiring. More interesting, altered tumor microenvironments are observed in hepatic metastases, especially a higher proportion of tumor infiltrating M2 macrophage and upregulation of complement cascade. Further experiments demonstrate that expression of C1q increases in primary tumors and hepatic metastases, C1q is mainly produced by M2 macrophage, and C1q promotes migration and invasion of PDAC cells.

**Conclusion:**

Taken together, we find potential factors that contribute to different stages of PDAC metastasis. Our study broadens the understanding of molecular mechanisms driving PDAC metastasis.

## Background

Pancreatic ductal adenocarcinoma (PDAC) is one of the most aggressive and lethal malignancies worldwide [1, 2]. Approximately 50% of newly identified PDAC patients are diagnosed with distant metastases, and the liver metastasis is the leading cause of death [3, 4]. So far, surgery remains the only curative treatment for pancreatic cancer. However, once distant metastases are diagnosed, the NCCN Clinical Practice Guidelines do not recommend radical surgical therapy, leading an extremely low 5-year survival rate (1%) with a median survival time of approximately 5.4–8.4 months [5]. To help development of effective treatment and improve survival, it is crucial to understand the molecular mechanisms of the hepatic metastasis of PDAC.

Metastasis is a complex multi-step process involving local invasion, intravasation, surviving in the blood circulation, extravasation, adapting to survival in new microenvironment, and finally colonization and outgrowth in distant body site [6]. However, our understanding in molecular mechanisms of tumor formation at the primary site of PDAC has far outpaced that in molecular traits of metastatic spread. Comprehensive genomic profiling of PDAC primary tumors revealed highly altered driver genes, such as *KRAS*, *TP53*, *CDKN2A*, and *SMAD4* [7–9]. Increase in dosage of *KRAS* mutant has been showed to drive early PDAC dissemination [10]. However, the heterogeneity of other driving genes in primary and metastatic tumors is limited [11, 12], indicating there are other factors driving PDAC metastasis. Transcriptomic characteristics of PDAC primary tumors have also been systematically studied [7, 9, 13]. Dysregulated processes such as epithelial to mesenchymal transition (EMT), morphological pattern formation, cancer stem cell regulation, and microenvironment remodeling [14–17] are critical for tumor cells to acquire metastatic capacity. Since metastatic PDACs are generally un-resectable based on the guidelines, there is little study concerning transcriptomic profile of metastatic lesion. A recent work revealed increased cell cycle in PDAC metastases by comparing primary PDACs to unpaired distant metastases [18]. However, there is not a study investigating transcriptomic profiles of matched primary tumor and hepatic metastasis of same PDAC patient. The transcriptomic features of PDAC metastases still remain largely unknown.

Growing evidences including our previous clinical study indicated synchronous surgery of hepatic oligometastasis and primary tumor would achieve encouraging survival with a median OS of 14.5–16.8 months in highly selective PDAC cases [19]. Here, for the first time, we systematically investigated the genomic and transcriptomic profiles of synchronous resected primary tumors (PTs), paired hepatic metastases (HMs), and primary tumor-adjacent normal pancreatic tissues (Ns) from 40 treatment-naïve (chemo- or radiotherapy) PDACs carrying hepatic oligometastasis. We sought to unravel the underlying mechanism of PDAC metastasis which would shed light on the development of novel therapeutic strategies for metastatic PDAC patients.

## Results

### Patients and study design

**Fig. 1:**
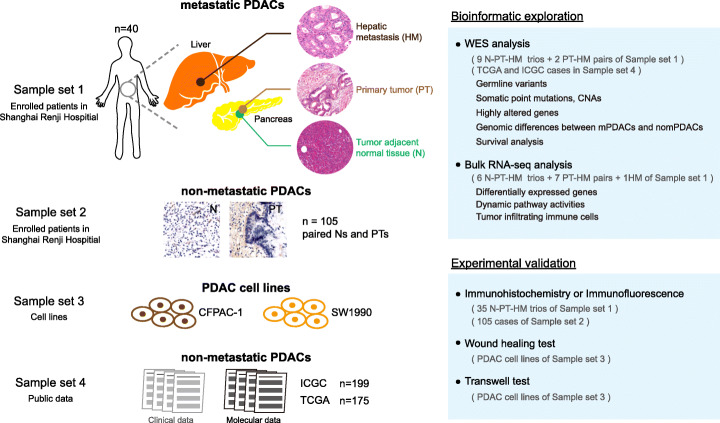
Study design and sample sets used in this study. Forty patients (sample set 1) were enrolled in this study. Genomic and transcriptomic profiling were performed to specimens from 17 patients of sample set 1. Experimental validation was performed using specimens from 35 patients of sample set 1, 105 non-metastatic patients from sample set 2, and two human PDAC cell lines from sample set 3. To explore the biological and clinical significance of identified events, molecular and survival data from previously published non-metastatic PDACs were used (sample set 4)

Forty treatment-naïve and synchronous surgically resected hepatic oligometastatic PDACs (Male, 25; Female, 15) were enrolled in this study (Fig. 1 sample set 1). The mean age of these patients is 62.2. Clinicopathological characteristics of all enrolled patients are provided in Additional file 1: Table S1. Genomic DNA of paired primary tumors (PTs) and hepatic metastases (HMs) from 11 patients (9 N-PT-HM trios and 2 PT-HM pairs) were assessed by WES. All tumor samples are of high purity (median 56.8%, Additional file 1: Figure S1A). Paired PTs and HMs from 13 patients (6 N-PT-HM trios and 7 PT-HM pairs) and one single HM specimen were evaluated by transcriptome sequencing. To validate our findings and explore their effects on PDAC metastasis, 35 N-PT-HM trios from sample set 1, specimens of 105 non-metastatic PDACs (105 N-PT pairs) from sample set 2, and two human PDAC cell lines in sample set 3 were used. Additionally, the molecular profiles and survival data of previously published non-metastatic PDAC cases (175 from TCGA [9], 199 from ICGC [7], Fig. 1 sample set 4) were used to investigate the biological and clinical significance of recurrently altered events of metastatic PDACs.

### Genetic heterogeneity between paired PTs and HMs revealed potential mechanisms of PDAC metastasis

**Fig. 2:**
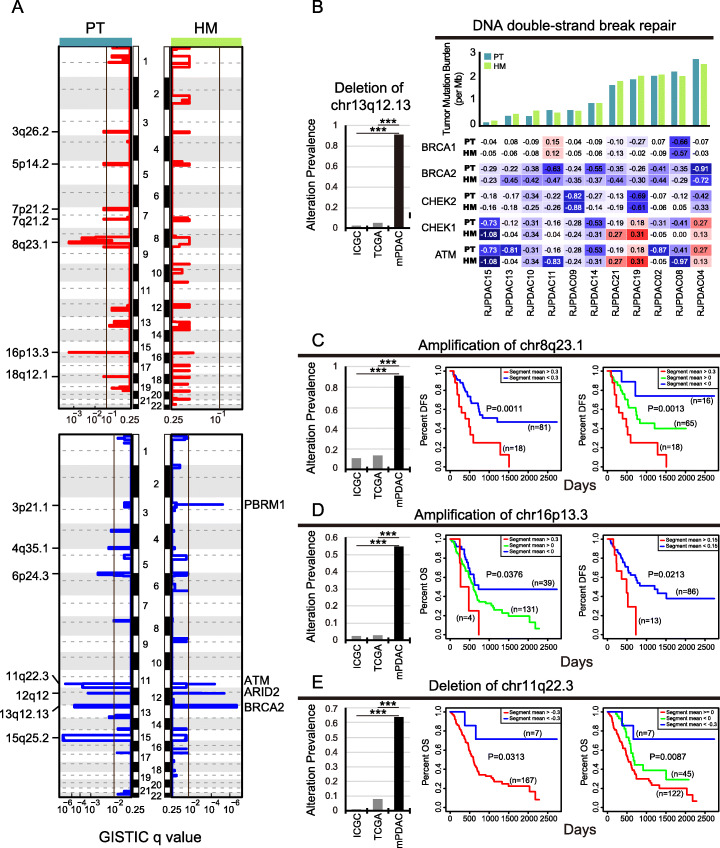
Potential clinical application of highly altered CNA events in hepatic metastatic PDAC. **a** Significantly occurred copy number amplifications (red, top panel) and deletions (blue, bottom panel) were identified in PT (left panel) and HM (right panel), respectively. Among them, deletion of 13q12.13 (**b**), amplification of 8q23.1 (**c**), amplification of 16q13.3 (**d**), and deletion of 11q22.3 (**e**) showed significantly higher alteration prevalence in metastatic PDACs (black bars in barplots) compared to non-metastatic PDACs (gray bars). ***P < 0.001. **b** Genes in chr13q12.13 contain BRCA2 that is a key component of DNA double-strand break repair pathway. Major components of this pathway showed recurrent CN loss in metastatic PDAC (right panel), suggesting these patients might be benefit from PPAR inhibitor. **c** Patients carrying CN gain of 8q23.1 are of higher metastatic risk. **d**, **e** CN amplification of 16q13.3 may serve as a biomarker for bad prognosis and higher metastatic risk while deletion of 11q22.3 may be used as a biomarker for good prognosis. As there was not enough case to test the association between DFS and gain of 16q13.3, we stratified patients according to a looser threshold. Patients with “segment_mean” of 16p13.3 greater than 0.15 were regarded as those with slight CN gain. DFS was defined as the interval between surgical treatment and date of diagnosis of distant metastases

We performed WES on specimens from 11 PDAC cases with hepatic oligometastasis. The average sequencing coverage of 129x and 63x were achieved on targeted regions for tumor samples and normal controls, respectively. In total, 1745 high-confident somatic point mutations (Additional file 2: Table S2) were identified which accounted for an average of 1.25/Mb tumor mutation burden (TMB) in PTs and an average of 1.22/Mb TMB in HMs. There is no significant difference of TMB between paired PTs and HMs (*P* > 0.1). Large proportions of somatic mutations identified in PTs were also present in paired HMs (ranged from 48 to 95.24%, Additional file 1: Figure S1B). In line with previous studies [7, 9, 11], PDAC driver genes such as *KRAS* (82%), *TP53* (63%), *CDKN2A* (45%), and *SMAD4* (45%) were highly mutated in enrolled patients (Additional file 1: Figure S1C). Somatic copy number (CN) amplification at 8q23.1 and 16p13.3 as well as CN deletion at 3p21.1, 6p24.3, 11q22.3, 12q12, 13q12.13, and 15q25.2 were recurrently occurred in hepatic oligometastatic PDACs (Fig. 2a and Additional file 3: Table S3).

Paired samples made it possible to compare the somatic mutation and copy number alterations (CNAs) of primary tumor to matched metastasis of individual patients. Consistent with previously studies [11], PTs and HMs showed limited heterogeneity in PDAC driver genes (Additional file 1: Figure S1C). *KRAS* mutations present in PTs were completely inherited by their corresponding HMs (Additional file 1: Figure S1C and Additional file 2: Table S2). Unlike mutations on driver genes, CN loss of 3p21.1 significantly occurred in HMs rather than in PTs (Fig. 2a). Comparing to paired PTs, HMs of several patients exhibited higher degree of 3p21.1 loss (Additional file 1: Figure S1D) suggesting a pre-existing clone harboring 3p21.1 deletion seeded the hepatic metastasis. Genes located at 3p21.1 encompass BAP1 and PBRM1 which involve in chromatin remodeling. Loss of 3p21.1 has been reported to precede metastasis of clean cell renal carcinoma [20, 21]. Deletion of BAP1 could facilitate metastasis of uveal melanoma [22, 23]. Moreover, studies on autopsy samples gathered from PDAC cases with multiple metastases also observed high frequency of CN deletion or loss of heterozygosity on 3p21.1 in their metastases [11, 12]. Therefore, we highly suspected that deletion of 3p21.1 was a potential mechanism of PDAC metastasis.

### Unique CNA signatures present in hepatic metastatic PDAC rather than non-metastatic cases imply great potential of clinical significance

Compared to non-metastatic PDACs, CNAs mentioned above were overrepresented in metastatic PDACs (Fig. 2b–e and Additional file 1: Figure S1E). For example, 10 of 11 sequenced cases carried deletion of 13q12.13 (91%) while there were only about 2–5% of non-metastatic PDACs harboring this CNA (Fig. 2b), indicating these highly altered CNAs were unique features of hepatic metastatic PDACs.

Genes located at 13q12.13 contain BRCA2 which plays an important role in homologous DNA repair pathway. It has been recently reported that PDAC patients with deficient homologous DNA repair system resulted from germline or somatic mutations on BRCA1 or BRCA2 are susceptible to Poly (ADP-ribose) polymerase (PARP) inhibitor therapy [24, 25]. However, cases harboring targetable mutations occur at quite low frequency (4–7%) [25]. To be noted, in our dataset, the two cases (RJPDAC04 and RJPDAC08) which showed most CN loss on BRCA genes were exactly the two with highest TMB (Fig. 2b). It suggested CN loss of BRCAs may also result in deficient DNA damage repair. As dysfunctional BRCAs offer possibility of targeted therapy for metastatic PDAC patients, this highlights the need to further investigate CN loss as candidate for loss-of-function event of BRCAs.

Additionally, some CNAs were of prognostic values. Patients with 8q23.1 gain tended to have worse disease-free survival (DFS). Moreover, the DFS decreased as the amplitude of 8q23.1 gain increased (Fig. 2c). Similar to findings in prostate cancer [26], amplification of chromatin 16p13.3 was associated with worse overall survival (OS) and worse DFS in PDAC (Fig. 2d). PDAC patients with deletion on 11q22.3 tended to have better OS (Fig. 2e). Thus, amplification of 8q23.1 and 16p13.3 may serve as biomarkers for predicting metastatic risk of PDAC while deletion of 11q22.3 may indicate better prognosis.

### HM possessed basic transcriptomic hallmarks of PDAC

**Fig. 3:**
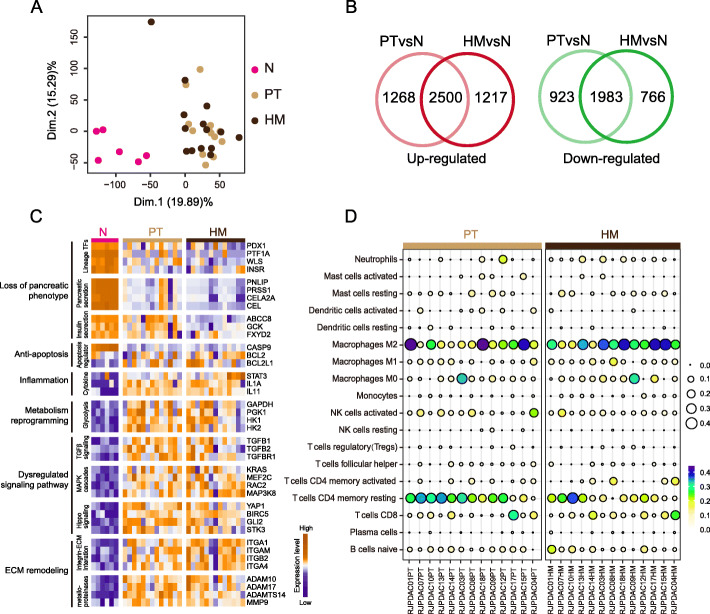
HMs bear basic transcriptomic hallmarks of PDAC. **a** Principal component analysis based on whole transcriptome showed that HMs have similar transcriptome as PTs. As showed in first two principal components that HMs and PTs are closed clustered together. **b** Compared to Ns, there are many common DEGs shared by PTs and HMs, including 2500 upregulated and 1983 downregulated genes. **c** Similar as PTs, HMs showed basic molecular hallmarks including loss of pancreatic phenotype, anti-apoptosis, inflammation, metabolic remodeling, dysregulated signaling pathways, and ECM remodeling. Genes showed in heatmap were those common DEGs identified in **b**. **d** PTs and HMs shared similar tumor-infiltrating-lymphocytes profiles

Although the genetic features of metastatic PDAC have been broadly explored, transcriptomic characteristics of human PDAC metastases remain largely unknown. Here, we conducted bulk RNA sequencing to 33 freshly frozen specimens of 14 PDACs with hepatic oligometastasis. As showed in principal component analysis, PTs and HMs were closely clustered together, while they were well separated from Ns (Fig. 3a). Up to 66% of differentially expressed genes (DEGs) between PTs and Ns were also identified as DEGs between HMs and Ns, including 2500 shared up-regulated genes and 1983 shared down-regulated genes (Fig. 3b, Additional file 1: Figure S2A). They involve in many biological processes such as loss of pancreatic functions, metabolic reprogramming, activation of PI3K-Akt signaling, and upregulation of cell migration (Fig. 3c, Additional file 1: Figure S2B).

On the other hand, HMs and PTs displayed higher expression level of genes involved in ECM remodeling and inflammation comparing to Ns (Fig. 3c). When referred to tumor ecosystem, both PTs and HMs displayed infiltration of T cells and macrophages (Fig. 3d). These findings indicated that both PTs and HMs comforted to the characteristics of desmoplasia which is a common trait of PDAC [27]. Collectively, PTs and HMs shared many common traits in their transcriptome.

### Hepatic metastases showed reverse of EMT and rewiring of metabolism comparing to primary tumors

It is worth noting that the extensive clinical heterogeneity with respect to treatment response [28] suggested there are substantial differences between PTs and HMs. Here, we studied the dynamic regulation pattern of gene expression across Ns, PTs, and HMs in pathway level.

**Fig. 4:**
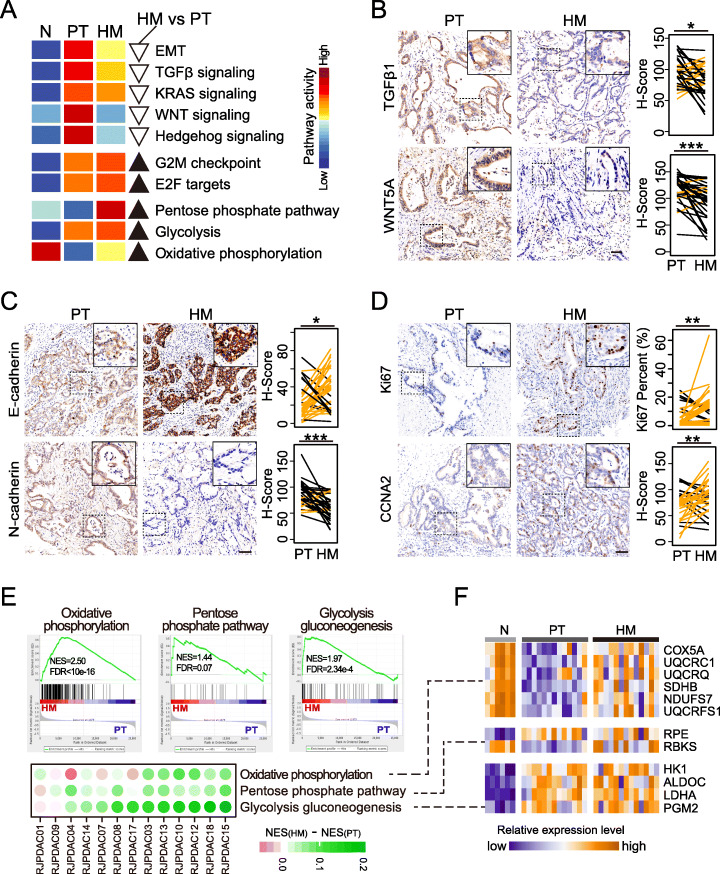
Reversed EMT and metabolism rewiring in HMs. **a** Compared to PT, HMs showed decreased activities of EMT and EMT related pathways (upper panel) as well as increased cell cycle activity (bottom panel). This phenomenon was experimentally validated by IFH assays of 35 paired PTs and HMs. **b** HMs showed significant upregulation of epithelial marker *E-cadherin* and downregulation of mesenchymal marker *N-cadherin*. **c** *TGFβ1* and *WNT5A* that are ligands of TGF*β* signaling and WNT signaling, respectively, were significantly upregulated in HMs. **d** Proliferative marker *Ki67* and *CCNA2* were upregulated in HMs compare to PTs. *0.01 < *P* < 0.05; **0.001 < *P* < 0.01; ***P < 0.001. **e** Oxidative phosphorylation, pentose phosphate pathway, and glycolysis were up-regulated in HMs. Top panel shows results of between-group comparisons. Bottom panel shows difference between HMs and PTs of individual cases. Green indicates up regulation while purple indicates down regulation in HM. **f** Gene expression heat-map of key components of pathways showed in **e**

We observed that oncogenic pathways were differentially regulated in PTs and HMs. For instances, TGFβ and WNT signaling were upregulated in both PTs and HMs comparing to Ns. However, when compared to PTs, these two pathways were downregulated in HMs (Fig. 4a). This partially reversed pattern was validated by immunohistochemistry (IHC) staining assays that *TGFβ1* (ligand of TGFβ signaling, *P* = 0.0280) and *WNT5a* (ligand of WNT signaling, *P* = 0.0002) were downregulated in HMs compared to corresponding PTs (Fig. 4b). Interestingly, EMT which is regulated by TGFβ signaling and WNT signaling displayed similar changing pattern as these two upstream pathways. The partially reversed EMT in HMs was verified by the upregulation of *E-cadherin* (*P* = 0.0003) and downregulation of *N-cadherin* (*P* = 0.0204) (Fig. 4c). To be noted, recent study about breast cancer revealed that the upregulation of *E-cadherin* could promote cell survival in circulation and tumor seeding in distant organ via the downregulation of TGFβ signaling [29]. Herein, we suspected that the upregulation of EMT in primary tumors would enhance their metastatic abilities while the reduced EMT activity in hepatic metastases would contribute to the colonization and outgrowth at new organ. Consistent with our postulate, we found the upregulation of G2M checkpoint and E2F targets in HMs (Fig. 4a). IHC assays of proliferation marker *ki67* (*P* = 0.0320) and *CCNA2* (*P* = 0.0056) confirmed the stronger proliferation of HMs (Fig. 4d).

Comparing to Ns, we found genes involved in oxidative phosphorylation pathway (OXPHOS) and pentose phosphate pathway (PPP) were inhibited in PTs while those involved in glycolysis were upregulated in PTs (Fig. 4a). The observation is consistent with previous studies that cancer cells prefer glycolysis rather than OXPHOS for ATP production even in the presence of oxygen (Warburg effect) [30]. That meets stringent bioenergetic demands of tumor cells as they are highly proliferative [31]. However, when compared to PTs, the depression of OXPHOS and PPP were partially reversed in HMs while the upregulation of glycolysis was further enhanced in HMs (Fig. 4a, e, and f). Interestingly, this phenomenon was also showed in breast cancer that, expression level of enzymes which played a role in glycolysis, OXPHOS, and PPP were increased in brain metastases compared to primary tumors [32]. To be noted, upregulated OXPHOS and PPP have been reported to be associated with enhanced proliferation of tumor cells [33, 34]. Given the stronger proliferation in HMs, we suspected the coexistence of these metabolic pathways would ensure the energy supply thus help tumor cells accommodate to new environment. Therefore, tumor cells would regulate pathway activities to better meet their needs in multi-step metastatic cascade.

### C1q acts in tumor microenvironment as a pro-metastasis factor

**Fig. 5:**
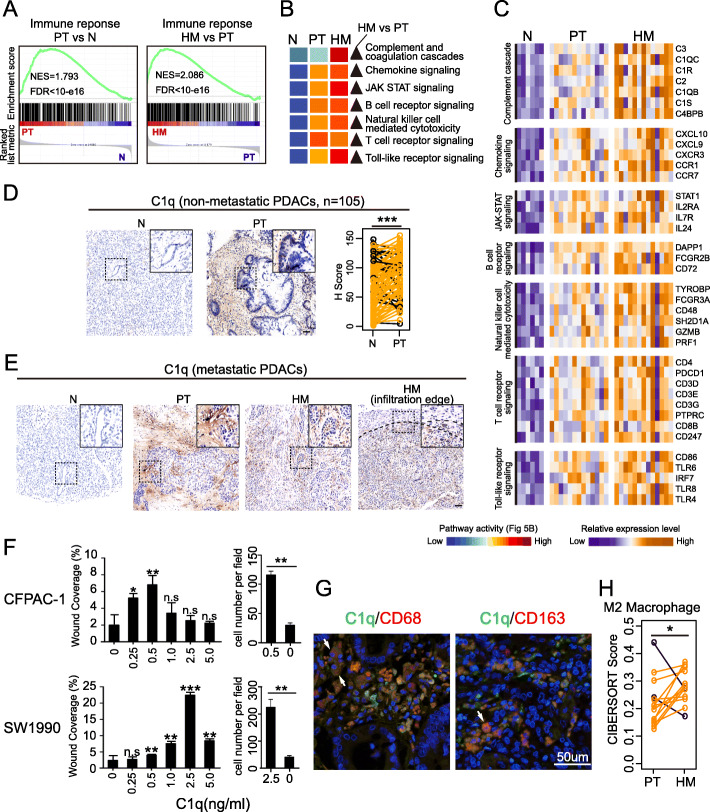
Tumor stroma plays important roles in PDAC metastasis. **a** Immune response was found gradually activated through N, PT, and HM. Comparing to Ns, the activity of immune response was increased in PTs (left panel). And it was further increased in HMs (right panel). **b** Multiple immune related pathways were found showing similar changing pattern as immune response. **c** Expression heatmap of representative genes of pathways showed in **b**. Among them, *C1q* that is involved in complement cascade was found mainly expressed at tumor stroma rather in tumor cells (**d** and **e**). Non-metastatic PDACs also showed overexpression of *C1q* in PTs compared to Ns (**d**). **f** Trans-well tests and wound healing test using PDAC cells lines CFPAC-1 and SW1990 showed that *C1q* would promote invasion and metastasis. **g** Identification of C1q-positive cells in PDAC. PDAC sections were double-stained for IF: *C1q* (green), *CD68* (macrophage marker, red; left panel), *CD163* (M2 macrophage marker, red; right panel). The double-positive cells appear in yellow (arrows). **h** In tumor microenvironment, M2 macrophages exhibited higher proportions in HMs compared to corresponding PTs

Besides tumor-cell intrinsic alterations, tumor microenvironment also displayed spatial heterogeneity between PTs and HMs. Comparing to Ns, PTs got stronger immune response which was further upregulated in HMs (Fig. 5a). The similar changing pattern was found in multiple immune-related pathways (Fig. 5b). Genes involved in these pathways were gradually upregulated across sample groups (Fig. 5c). Among these pathways, chemokine signaling [35], JAK-STAT signaling [36, 37], B cell receptor signaling [38, 39], T cell receptor signaling [17], Toll-like receptor signaling [40], and natural killer cell-mediated cytotoxicity [41] have been broadly investigated in initiation, progression, or metastasis of PDAC. However, the contribution of complement system to metastasis of PDAC still remains unclear.

Complement cascade is triggered by three mechanisms: classical pathway, lectin pathway, and alternative pathway. Genes coding key components of classical pathway were stepwise unregulated across sample groups (Fig. 5c), including those coding *C1q* (*C1QB*, and *C1QC*) that serves as a recognition and regulatory protein of classical pathway. Interestingly, the overexpression of *C1q* was emerged as early as tumor cells had not metastasized. In 105 non-metastatic PDACs, we found *C1q* was significantly upregulated in primary tumors than paired normal controls (Fig. 5d). It suggested overexpression of *C1q* was an early event and contributes to tumor metastasis.

To be noted, *C1q* is the only complement of classical pathway that expressed in tumor stroma of both PTs and HMs (Fig. 5d, e, Additional file 1: Figures S3 and S4A). Additionally, compared to normal liver tissues, *C1q* was overexpressed at HM adjacent normal livers (Additional file 1: Figure S5). These implied *C1q* might be involved in formation of tumor-friendly microenvironment. Moreover, *C1q* could be clearly detected at the tumor infiltration edge in HMs (Fig. 5d) suggesting it might play a role in tumor invasion and metastasis. In vitro experiments proved that exogenous purified human C1q could promote migration and invasion of PDAC cells (Fig. 5f). Thus, *C1q* acts in tumor environment might be a potential mechanism of PDAC metastasis.

To further explore the role of *C1q* in PDAC metastasis, we investigated the resources of *C1q*. According to previous study which evaluated PDAC tumor ecosystem at single-cell resolution [42], most of sequencing reads mapped on *C1QA*, *C1QB*, and *C1QC* were contributed by macrophage (Additional file 1: Figure S4B), demonstrating macrophage might be the main resource of *C1q*. Macrophage is among the most abundant tumor infiltrating immune cells (Fig. 3d), which exhibited elevated relative proportions in HMs compared to corresponding PTs (*P* = 0.003, Fig. 5h). By immunofluorescence assays, we found *C1q* was co-localized with macrophage (*CD68*), especially M2 macrophage (*CD163*) (Fig. 5g, Additional file 1: Figure S4A). Herein, M2 macrophage-derived *C1q* in tumor microenvironment might act as a metastasis-promoting factor in PDAC.

## Discussion

**Fig. 6:**
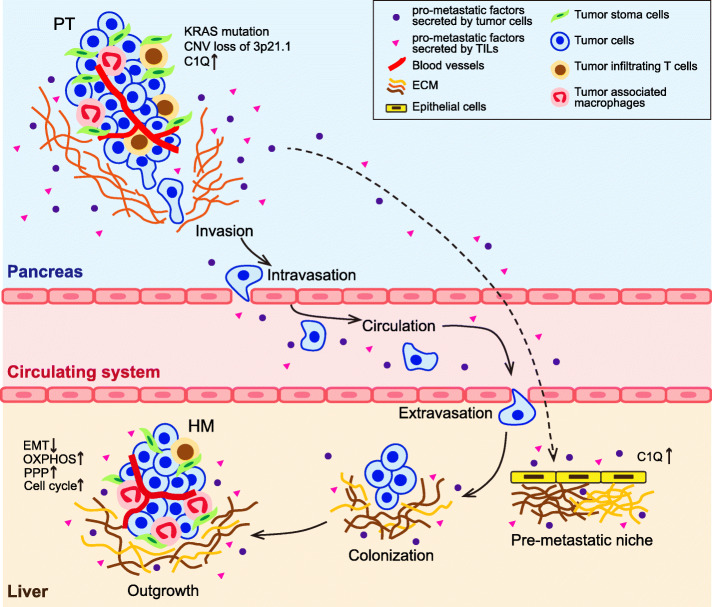
Schematic plot of multi-step hepatic metastasis of PDAC. To successfully build a second tumor clone, primary tumor cells of PDAC must go through a series of steps including local invasion, intravasation, surviving in the circulation system, extravasation, adapting to survival in new microenvironment, colonization, and outgrowth in liver. This figure was adapted from [43]. The types of cell that presented in tumor microenvironments and pro-metastatic factors marked in the figure were modified according to our findings

This study systematically investigated the molecular profiles of synchronous resected PTs and HMs from 40 hepatic oligometastatic PDAC patients. We observed concordance in genomic and transcriptomic hallmarks between HMs and paired PTs. And we deciphered the molecular heterogeneities between paired PTs and HMs both in tumor cells and tumor microenvironments. The significantly occurred deletion of 3p21.1 in HMs rather than PTs reversed EMT process as well as reprogramed metabolism in HM, and overexpression of *C1q* at tumor microenvironment could enhance tumor cell invasion and migration (Fig. 6). Moreover, by compared to non-metastatic PDACs, some CNAs were of great clinical potential. These findings provide new authentic insights into molecular mechanisms of hepatic metastases of PDAC that may have considerable implications for the prognosis and precise medical treatment of PDAC patients bearing oligometastasis.

Resected specimens used in our study offer great chance to systematically depict genetic and transcriptomic features of human PDAC metastases, which cell lines, biopsy specimens, genetically engineered mouse models, patient-derived xenografts, or autopsy specimens [7, 11, 44, 45] are not sufficient to unravel. Additionally, we attached great importance to investigate treatment-naïve cases, as chemotherapy and target therapy could alter genetic and transcriptomic features of tumor cells as well as the compositions of tumor microenvironment [46, 47]. Moreover, synchronously resected paired PTs and HMs provide unique opportunity to investigate both initial and end stage of metastatic cascade. Thus, without external interventions, our specimens allowed us to explore the true nature of how PDAC evolves.

The metastases spawned by carcinomas are formed following the completion of a complex succession of cell-biological events including local invasion, intravasation into blood vessels, survival in circulation, extravasation, survival in foreign microenvironments, and outgrowth at the metastatic site (Fig. 6) [6, 43]. Only a small fraction of cells disseminated from primary tumor could enter the circulation, and 0.01% of these could successfully establish metastases [48]. Through our investigation, we thought that PDAC tumor cells could interconvert between various strategies to best meet their requirements in every step of metastatic cascade. In the early phase, *KRAS* mutation, CN loss of 3p21.1, and upregulation of EMT facilitate dissemination from primary tumor. However, when tumor cells arrive at distant body locations, the top priority shift from dissemination to survival, colonization, and further outgrowth in new environment. To this end, disseminated tumor cells downregulated EMT in late phase of metastasis, to re-acquire epithelial phenotype (up-regulation of *E-cadherin*). Study in breast cancer observed similar phenomenon that *E-cadherin* inhibited local invasion but promote metastatic colonization while its depletion would reduce colony formation at distant organ [29]. In addition, we observed that OXPHOS, PPP, and glycolysis were upregulated in distant metastases, demonstrating tumor cells in hepatic metastasis adopt distinct metabolism fashion as those in primary tumor. Altered glucose metabolism is often linked to highly proliferative phenotype in tumor. For instance, *C-MYC* can concurrently drive aerobic glycolysis and OXPHOS according to the tumor cell microenvironment [49]. As a major contributor to anabolism, the PPP has been well-described to support tumor cell proliferation [34], which displayed an enhanced activity in brain metastases of breast cancer compared to circulating tumor cells [32] and in metastatic lesions compared to primary tumors in renal cell carcinoma [50]. Thus, rewiring of metabolism in HMs might ensure the energy supply for the highly proliferative tumor cells thus better accommodate and outgrowth at distant organ.

Except the tumor cell intrinsic features, factors generated from tumor environment also play crucial roles in PDAC metastasis [17]. Our analysis showed HMs had higher percentage of macrophage M2 that was previously related to worse survival of PDAC patients [51]. We also confirmed that multiple immune-related pathways, such as complement and coagulation cascades, remarkably activated in HMs compared to PTs. Furthermore, we demonstrated the M2 macrophage-derived complement *C1q* acting in tumor microenvironments would promote invasion and migration of pancreatic cancer cell. To be noted, accumulation of *C1q* would induce systematic inflammation and further recruit tumor-associated immune cells [52, 53]. Those are characteristics defining pre-metastatic niche [53]. Given the observation that upregulation of *C1q* was an early event in metastatic process as well as it was overexpressed in HM adjacent liver tissues compared to normal liver, we suspected that the hostile milieu of the liver was selectively preconditioned by *C1q* at an early stage to render liver more conducive to the engraftment and growth of disseminated PDAC cells (Fig. 6). However, further investigations are needed to unravel the detailed mechanisms of C1q in the metastatic cascade of PDAC.

Most importantly, these findings have great clinical significance. Synchronous surgery of liver metastasis and primary tumor is theoretically considered as a treatment that has potential to offer improved survival and better life quality for PDAC patients with hepatic oligometastases. However, resection of hepatic oligometastasis is still a controversial issue. Re-acquired epithelial phenotype of HMs implied colonization and outgrowth rather than further metastasis were the top priority of PDAC cells in distant organ. This point of view was corroborated by Nicole and colleagues who showed metastatic cells appear to re-acquire an epithelial phenotype with increasing lesion size in both mouse and human PDAC [44]. The lower metastatic but higher proliferative capabilities of HMs suggested the theoretical feasibility of surgical synchronous resection of HMs and PTs in metastatic PDAC. Additionally, IHC staining indicated that CD8+ T cell (*CD8A*) were more abundant in HMs comparing to corresponding PTs (*P* = 0.006, Additional file 1: Figure S6A), suggesting the possibility of immune therapy of PDAC cases with oligometastases. Moreover, we found the upregulation of tumor immunity in HMs is accompanied with the downregulation of NOTCH signaling (Additional file 1: Figure S6B). NOTCH signaling has been reported playing an immunosuppressive role in tumor progression [54] which suggested combination of NOTCH inhibitor and immune therapy might achieve better clinical efficacy. In addition, unique CNA signatures of hepatic metastatic PDAC suggested genomic gain at 16p13.3 as well as 8q23.1 may act as biomarkers indicating higher metastatic risk in PDAC.

## Conclusion

To the best of our knowledge, this is the first study that comprehensively explores the genomic and transcriptomic profiles of synchronous resected PTs and matched HMs from treatment-naïve oligometastatic PDACs. Factors that contribute to metastasis may emerge at the any stage of the cascade. Genetic mutations of *KRAS*, copy number deletion of 3p21.1, and activated EMT process enhance the metastatic ability of primary tumor cells. And primary tumor might educate tumor infiltrating immune cells to secrete pro-metastatic factors (such as *C1q*) to help pre-metastatic niche formation in the liver. When tumor cells arrive at liver, tumor cells might remodel energy metabolism (upregulation of OXPHOS and PPP program) and regulate their gene expression (downregulation of EMT process) to better meet their demands at new microenvironments.

This study broadens our understandings of mechanisms of PDAC metastasis. Our findings are of great clinical relevance. Identification of factors that predict metastatic risk would help stratify patients of different metastatic risk. That would help optimize clinical treatment of pancreatic cancer. Moreover, the lower metastatic but higher proliferative capabilities of HM provide theoretical feasibility of surgical synchronous resection of HMs and PTs of hepatic oligometastatic PDAC.

## Methods

### Sampling and DNA extraction

Samples were acquired basing on our previous clinic study. All the samples were collected with documented informed consents from the enrolled patients. The normal liver specimens were provided by Professor Li Jun (State Key Laboratory of Oncogenes and Related Genes, Shanghai Cancer Institute, Renji Hospital, Shanghai Jiao Tong University School of Medicine). The tissue taken for analysis was enriched for tumor cells after the evaluation of hematoxylin and eosin (H&E)-stained slides by a pathologist and another gynecologic pathologist to confirm histological diagnosis. Based on this evaluation, 2.0- or 2.5-mm tissue punches were taken from the selected tumor foci in the FFPE block using a tissue microarrayer (Beecher Instruments, Sun Prairie, WI, USA). DNA was extracted with QIAamp DNA Mini Kit according to the manufacturer (Qiagen, Germany). The DNA sample quality and integrity were analyzed by A260/280 ratio and agarose gel electrophoresis using Qubit 2.0 (Life Technologies, USA) and Nanodrop 2000 (ThermoScientific, USA).

### Whole-exome sequencing

For library preparation, 1 μg genomic DNA were sheared to fragments of ~ 250 bp by Covaris’ ultrasonicator (Covaris, Germany), then end-repaired, A-tailed and ligated to Illumina sequencing adapters. The ligated products were size selected 300~400 bp on AMpure XP beads (Beckman, Germany) and amplified by LM-PCR. The amplified samples were hybridized to capture exon/target regions by SeqCap EZ Human Exome Library v3.0 (Roche, Sweden) for at least 20 h at 42 °C. After hybridization, the captured products were purified by Dynabeads® M-270 Streptavidin (Life Technologies, USA), then amplified by KAPA HiFi PCR Master Mix (KAPA Biosystem, USA). The libraries were sequenced on Illumina Hiseq X-Ten with 2 × 150 bp paired-end sequencing, which were controlled by Hiseq Control Software (HCS).

### Whole-exome sequencing data processing

FASTQC was used to assess the quality of raw sequencing reads. Bad quality reads or bases were removed. Then, the clean reads were mapped to human reference genome (hg19) using BWA-MEM algorithm (v0.7.12) [55]. Duplicated reads were marked for filtering using Picard MarkDuplicates (v2.3). INDELs were realigned using GATK (v3.5) [56] IndelRealigner and the base quality scores were recalibrated using GATK BaseRecalibrator. The resulting BAM files were used to identify germline and somatic alterations.

### Germline and somatic mutations calling

Germline variants were generated by GATK HaplotypeCaller. To identify high confident somatic mutations, we combined the results of multiple variation calling tools. MuTect (v1.1.7) [57], VarScan2 (v2.3.9) [58], and Strelka (v2.7.1) [59] were employed to call somatic mutations. For INDELs, VarScan2, lofreq (v2.1.2) [60], and Strelka were used. Only high-quality variations that identified by at least two methods were considered as candidates of somatic mutations for individual samples. For tumors without normal controls, we generated a reference list consisting of all germline alterations of all normal samples. Only alterations that are other than those in the reference list were considered candidates of somatic mutation for these samples. All candidates were further annotated using Oncotator (hg19) [61]. INDELs present in repeat regions were removed. Mutations with allele frequency greater than 0.1% in 1000 Genome Project were removed. The resulting mutations were regarded as somatic mutations for individual tumor samples.

### Copy number alteration calling and tumor purity

Somatic CNAs were calculated by CNVkit (v0.9.1) [62] which used a pooled reference of per-bin copy number estimates from all normal samples. Regions with |log2ratio| value greater than 0.3 were regarded as CNA regions. To further confirm that sequenced tumor samples are those of relative high tumor purity, the outcomes of CNVkit were used as input for ABSOLUTE algorithm [63] to infer tumor purity and ploidy. In consistent with our previous operations (enrichment of tumor cells), ABSOLUTE showed that our tumor samples are of high tumor purity (Additional file 1: Figure S1A). Significantly altered CNA regions were informed using GISTIC2 algorithm [64].

### Comparisons with non-metastatic PDACs and survival analysis

We retrieved survival data and public accessible copy number segment file of non-metastatic pancreatic ductal adenoma were from TCGA and ICGC. Both segment files were generated from SNP array data. And germline alterations had already been marked. Regions with |log2ratio| value (segment mean) greater than 0.3 were regarded as CNA regions. To compare occurrence rate of CNA events that were highly altered in our metastatic PDACs to those of non-metastatic cases, regions with at least 10% overlapped were compared. One-sided fisher’s exact tests were used to test if individual CNA events had increased occurrence rate in metastatic PDAC than in non-metastatic cases. *P* value less than 0.05 was considered significant.

To further explore whether these CNA events were associated with overall survival or metastatic risk, we used R package “survival” [65] to test whether there were differences in OS or DFS between patient groups. DFS was defined as time interval between surgical treatment and the date of diagnosed of distant metastasis. Univariate cox regressions were used to calculate *p* value. P value less than 0.05 was considered significant.

### RNA sequencing

Total RNA was prepared with QIAzol Lysis Reagent and RNeasy MinElute Cleanup Kit (Qiagen, Germany). The RNA quality and integrity were analyzed by Qubit 2.0(Life Technologies, USA) and Bioanalyzer 2100 (Agilent, Germany). For library preparation, 3 μg total RNA were captured by NEBNext Oligo d(T) 25 beads (NEB, USA), sheared to fragments of ~ 250 bp, and reverse transcripted by NEBNext RNA first and second Strand Synthesis Module (NEB, USA). The products were end-repaired, A-tailed, and ligated to Illumina sequencing adapters and amplified by PCR. The sequencing library were qualified by Qubit 2.0 (Life technologies, USA) and Bioanalyzer 2100 (Agilent, Germany), then sequenced on Illumina Hiseq X-Ten with 2 × 150 bp paired-end sequencing, which were controlled by Hiseq Control Software (HCS).

### RNAseq data processing

The sequencing quality of raw reads was firstly assessed with FASTQC. Next, the trimGalore (v0.5.0) were used to trim bad-quality bases or reads. The resulting clean reads were then mapped to human genome reference (hg19) by using STAR (v2.5.2b) algorithm [66]. After that, the htseq-count (v0.6.0) [67] were used to count the total number of uniquely mapped reads mapped to each gene. The raw count data was further normalized by size factor described as previous work [68].

### PCA, differential expressed genes, and gene set enrichment analysis

Genes with at least 5 normalized counts in at least 90% of all samples were used to perform principal component analysis by FactoMineR R package [69]. DESeq2 R package [70] was used to call differential expressed genes (DEGs) between two groups. Genes with adjust *p* value (FDR) < 0.05 and |log2(fold change)| > 1 were considered as DEGs. Function annotation and pathway enrichment of DEGs were performed using DAVID [71]. Unsupervised hierarchical clustering was conducted on log2-tranformed normalized counts to explore expression pattern across normal tissues, primary tumors, and distant metastases. Gene Set Enrichment Analysis (GSEA) was employed to detect gene sets that show significant differences between two given groups. Gene sets with FDR values less than 0.25 were considered as significantly different. Gene set variation analysis (GSVA) [72] was used to conduct pathway analysis at single-sample level.

### Deciphering the proportion of tumor infiltrating immune cells

We employed both CIBERSORTx [73] and xCell algorithms [74] to estimate the abundance of tumor infiltrating immune cells. For a specific sample, we identified top abundant types of immune cells within TME by using CIBERSORTx in “relative” mode which allows between-cell type comparisons [75]. As xCell is capable of deciphering dozens of types of immune cells and the result of which is allowed for between-sample comparison [75], we identified differentially infiltrated immune cells between paired PTs and HMs using the results of xCell. The xCell algorithm that packed in immundeconv R package [76] is used. TPM (transcript per million) matrix is used as input for both CIBERSORTx and xCell. One-sided Wilcox rank sum test is used for testing whether a specific type of immune cell is more (or less) abundant in HMs compared to paired PTs.

### Correlation between oncogenic signatures with tumor infiltrating immune signatures

To explore the association between oncogenic pathways and tumor immunity during PDAC metastasis, we firstly identified oncogenic pathways that are significantly correlated with immune-related signatures. Correlations between oncogenic pathways and immune-related pathways were measured by Spearman correlation between NES values (GSVA) of the former and those of the latter. Correlations between oncogenic pathways and relative proportions of immune cells were calculated as Spearman correlations between NES values of oncogenic pathways and xCell scores of tumor infiltrating immune cells.

### Tissue microarray (TMA) construction

Tissue microarrays (TMA) were constructed using diameter of 1.5-mm cores including 35 cases of matched HMs, PTs, and non-tumor tissues specimens. Twelve of 35 patients were our sequencing patients. After screening and marking representative spots of tissues, the tissues were punched out and squeezed into the paraffin array blocks.

### Immunohistochemistry

Immunohistochemical staining was performed on a TMA and paraffin sections at 4-μm thickness as previously described. An intensity score of 0 to 3 was assigned for the intensity of tumor cells (0, none; 1, weak; 2, intermediate; 3, strong). A proportional score was given by the estimated proportion of positive tumor cells in percentage. To assess the average degree of staining within a tumor, multiple regions were analyzed, and at least 100 tumor cells were assessed. The cytoplasmic expression was assessed by H-score system. The formula for the H-score is Histoscore = ∑(I × Pi), where I = intensity of staining and Pi = percentage of stained tumor cells, producing a cytoplasmic score ranging from 0 to 300. We implemented immunohistochemistry for E-cadherin (Servicebio, GB13083), Cyclin A2 (Abcam, ab181591), N-cadherin (Servicebio, GB13136), ki67 (Servicebio, GB13030-2), Wnt5a (Proteintech, 55184-1-AP), TGFβ1 (Servicebio, GB11179) on TMA and C1q (Dako, Code A0136), CD68 (Abcam, ab955), CD163 (Abam, ab87099), CD8A (Abam, ab93278). DAPI (Life Technologies, 62247) was used as a nuclear counterstain. Images were obtained using the Zeiss Axioplan 2 Fluorescence microscope.

## Supplementary Information

**Additional file 1: Table S1.** Clinicopathological characteristics of all enrolled metastatic patients; **Figure S1.** Mutation profiles of paired PTs and HMs; **Figure S2.** Expression pattern and enriched pathways of common DEGs; **Figure S3.** IHC staining of key components of classical complementary pathways in PT and HM specimens; **Figure S4.** IF staining of C1q, macrophage cell marker and epithelial cell marker in tumor specimens as well as single-cell RNAseq data analysis of the resources of C1Q. **Figure S5.** IHC staining of key components of classical complementary pathways in HM adjacent liver tissue and normal liver tissue. **Figure S6.** Correlation of oncogenic pathways and tumor immunity; supplementary figure legend and supplementary table notes.

**Additional file 2: Table S2.** Somatic mutation list.

**Additional file 3: Table S3.** Significantly altered CNA regions.

**Additional file 4.** Review history.

## Data Availability

Raw sequencing data of whole-exome sequencing and RNA-seq were deposited in European Genome-phenome Archive (EGA) with the accession code EGAD00001006599 [77] and EGAD00001006598 [78], respectively. These data would also be accessed in National Omics Data Encyclopedia (NODE) database with project ID OEP000481 [79]. Raw count table was deposited in Gene Expression Omnibus (GEO) database with the accession number GSE151580 [80]. Clinical and molecular data of non-metastatic PDACs deposited at TCGA and ICGC are retrieved from GDC data portal (https://portal.gdc.cancer.gov/projects/TCGA-PAAD) [9] and ICGC data portal (https://dcc.icgc.org/projects/PACA-AU) [7].
