# Supplementary material for: Integrated genomic and transcriptomic analysis reveals unique characteristics of hepatic metastases and pro-metastatic role of complement C1q in pancreatic ductal adenocarcinoma
Source: Genome Biol. 2021 Jan 4;22:4. doi: 10.1186/s13059-020-02222-w (PMC7780398; doi:10.1186/s13059-020-02222-w)
Supplement: Supplementary file 1 — Additional file 1: Table S1. Clinicopathological characteristics of all enrolled metastatic patients; Figure S1. Mutation profiles of paired PTs and HMs; Figure S2. Expression pattern and enriched pathways of common DEGs; Figure S3. IHC staining of key components of classical complementary pathways in PT and HM specimens; Figure S4. IF staining of C1q, macrophage cell marker and epithelial cell marker in tumor specimens as well as single-cell RNAseq data analysis of the resources of C1Q. Figure S5. IHC staining of key components of classical complementary pathways in HM adjacent liver tissue and normal liver tissue. Figure S6. Correlation of oncogenic pathways and tumor immunity; supplementary figure legend and supplementary table notes. [file 13059_2020_2222_MOESM1_ESM.pdf]

# Additional File 1 for

## Integrated genomic and transcriptomic analysis reveals unique characteristics of hepatic metastases and pro-metastatic role of complement C1q in pancreatic ductal adenocarcinoma

Jianyu Yang[1*], Ping Lin[2*], Minwei Yang[1*], Wei Liu[1], XueLiang Fu[1], DeJun Liu[1], LingYe Tao[1], YanMiao Huo[1], JunFeng Zhang[1], Rong Hua[1], ZhiGang Zhang[3#], YiXue Li[2,4,5,6#], Liwei Wang[3,7#], Jing Xue[8#], Hong Li[2#], Yongwei Sun[1#]

1. Department of Biliary-Pancreatic Surgery, Ren Ji Hospital, School of Medicine, Shanghai Jiao Tong University, Shanghai 200127, China.
2. CAS-MPG Partner Institute for Computational Biology, Shanghai Institute of Nutrition and Health, Shanghai Institutes for Biological Sciences, University of Chinese Academy of Sciences, Chinese Academy of Sciences, Shanghai 200031, PR China.
3. State Key Laboratory of Oncogenes and Related Genes, Shanghai Cancer Institute, Ren Ji Hospital, School of Medicine, Shanghai Jiao Tong University, Shanghai 200240, China.
4. School of Life Sciences and Biotechnology, Shanghai Jiao Tong University, Shanghai 200240, China.
5. Collaborative Innovation Center for Genetics and Development, Fudan University, Shanghai, 200032, China.
6. Shanghai Center for Bioinformation Technology, Shanghai Academy of Science & Technology, Shanghai, 201203, China.
7. Department of Oncology, Ren Ji Hospital, School of Medicine, Shanghai Jiao Tong University, Shanghai 200127, China.
8. State Key Laboratory of Oncogenes and Related Genes, Renji-Med X Clinical Stem Cell Research Center, Shanghai Cancer Institute, Shanghai Jiao Tong University School of Medicine Affiliated Renji Hospital, Shanghai 200240, China.

**[#]Correspondence to:** Yongwei Sun (E-mail: syw0616@126.com); Hong Li ( E-mail: lihong01@sibs.ac.cn); Yixue Li (E-mail: yxli@sibs.ac.cn); Jing Xue (E-mail: xuejing0904@126.com); Liwei Wang (E-mail: liweiwang@shsmu.edu.cn) and Zhigang Zhang (E-mail: zzhang@shsci.org)

*** Authors contributed equally to this work**

## Contents:

Table S1

Fig. S1-S6

Supplementary Table Notes

Supplementary Figure Legends

Reference

Table S1. clinicopathological characteristics of all enrolled metastatic patients

| Patient | Tumor location | Sex | Age (years ) | Treatmen t before surgery | Primary tumor histologic type of | Survival (month) | WES | | | RNA-seq | | | Validation |
|---|---|---|---|---|---|---|---|---|---|---|---|---|---|
| | | | | | | | N | T | HM | N | T | HM | N-T-HM |
| RJPDAC01 | Body/Tail | M | 57 | NO | PDAC | 56 | X | X | X | √ | √ | √ | √ |
| RJPDAC02 | Body/Tail | F | 59 | NO | PDAC | - | √ | √ | √ | X | X | √ | √ |
| RJPDAC03 | Body/Tail | M | 59 | NO | PDAC | 13.5 | X | X | X | X | √ | √ | √ |
| RJPDAC04 | Body/Tail | F | 62 | NO | PDAC | 20.8 | √ | √ | √ | X | √ | √ | √ |
| RJPDAC07 | Head | F | 48 | NO | PDAC | 4.1 | X | X | X | X | √ | √ | X |
| RJPDAC08 | Body/Tail | M | 68 | NO | PDAC | 7.3 | √ | √ | √ | √ | √ | √ | √ |
| RJPDAC09 | Body/Tail | F | 59 | NO | PDAC | 6.2 | √ | √ | √ | X | √ | √ | √ |
| RJPDAC10 | Body/Tail | F | 67 | NO | PDAC | 16.5 | √ | √ | √ | √ | √ | √ | √ |
| RJPDAC11 | Body/Tail | M | 59 | NO | PDAC | 6.2 | √ | √ | √ | X | X | X | √ |
| RJPDAC12 | Head | M | 75 | NO | PDAC | 17.1 | X | X | X | √ | √ | √ | X |
| RJPDAC13 | Body/Tail | F | 53 | NO | PDAC | 17.5 | √ | √ | √ | √ | √ | √ | √ |
| RJPDAC14 | Body/Tail | M | 57 | NO | PDAC | 16.1 | √ | √ | √ | X | √ | √ | √ |
| RJPDAC15 | Body/Tail | M | 63 | NO | PDAC | 7.8 | √ | √ | √ | √ | √ | √ | √ |
| RJDPAC17 | Head | F | 64 | NO | PDAC | 6.0 | X | X | X | X | √ | √ | √ |
| RJPDAC18 | Head | M | 66 | NO | PDAC | 6.0 | X | X | X | X | √ | √ | X |
| RJPDAC19 | Head | M | 68 | NO | PDAC | 2.2 | X | √ | √ | X | X | X | X |
| RJPDAC21 | Body/Tail | F | 66 | NO | PDAC | 3.9 | X | √ | √ | X | X | X | X |
| RJPDAC22 | Head | F | 60 | NO | PDAC | 31.0 | X | X | X | X | X | X | √ |
| RJPDAC23 | Head | M | 55 | NO | PDAC | 4.3 | X | X | X | X | X | X | √ |
| RJPDAC24 | Head | M | 71 | NO | PDAC | 17.0 | X | X | X | X | X | X | √ |
| RJPDAC25 | Head | F | 82 | NO | PDAC | 5.2 | X | X | X | X | X | X | √ |
| RJPDAC26 | Head | M | 40 | NO | PDAC | 3.6 | X | X | X | X | X | X | √ |
| RJPDAC27 | Head | F | 50 | NO | PDAC | 19.0 | X | X | X | X | X | X | √ |
| RJPDAC28 | Head | F | 68 | NO | PDAC | 4.1 | X | X | X | X | X | X | √ |
| RJPDAC29 | Head | M | 58 | NO | PDAC | 5.7 | X | X | X | X | X | X | √ |
| RJPDAC30 | Head | M | 58 | NO | PDAC | 8.9 | X | X | X | X | X | X | √ |
| RJPDAC31 | Body/Tail | M | 63 | NO | PDAC | 14.5 | X | X | X | X | X | X | √ |
| RJPDAC32 | Body/Tail | M | 65 | NO | PDAC | 7.7 | X | X | X | X | X | X | √ |
| RJPDAC33 | Body/Tail | M | 69 | NO | PDAC | 15.0 | X | X | X | X | X | X | √ |
| RJPDAC34 | Body/Tail | F | 69 | NO | PDAC | 16.9 | X | X | X | X | X | X | √ |
| RJPDAC35 | Body/Tail | M | 75 | NO | PDAC | 21.0 | X | X | X | X | X | X | √ |
| RJPDAC36 | Body/Tail | M | 47 | NO | PDAC | 21.0 | X | X | X | X | X | X | √ |
| RJPDAC37 | Body/Tail | M | 69 | NO | PDAC | 22.0 | X | X | X | X | X | X | √ |
| RJPDAC38 | Head | F | 69 | NO | PDAC | 1.0 | X | X | X | X | X | X | √ |
| RJPDAC39 | Head | M | 64 | NO | PDAC | 7.8 | X | X | X | X | X | X | √ |
| RJPDAC40 | Body/Tail | M | 58 | NO | PDAC | 2.8 | X | X | X | X | X | X | √ |
| RJPDAC41 | Body/Tail | F | 58 | NO | PDAC | 3.2 | X | X | X | X | X | X | √ |
| RJPDAC42 | Body/Tail | M | 53 | NO | PDAC | 8.2 | X | X | X | X | X | X | √ |
| RJPDAC43 | Body/Tail | M | 61 | NO | PDAC | 9.7 | X | X | X | X | X | X | √ |
| RJPDAC44 | Body/Tail | M | 77 | NO | PDAC | 17.0 | X | X | X | X | X | X | √ |
| | Total | | | | | | 9.00 | 11.00 | 11.00 | 6.00 | 13.00 | 14.00 | 35.00 |

Fig S1

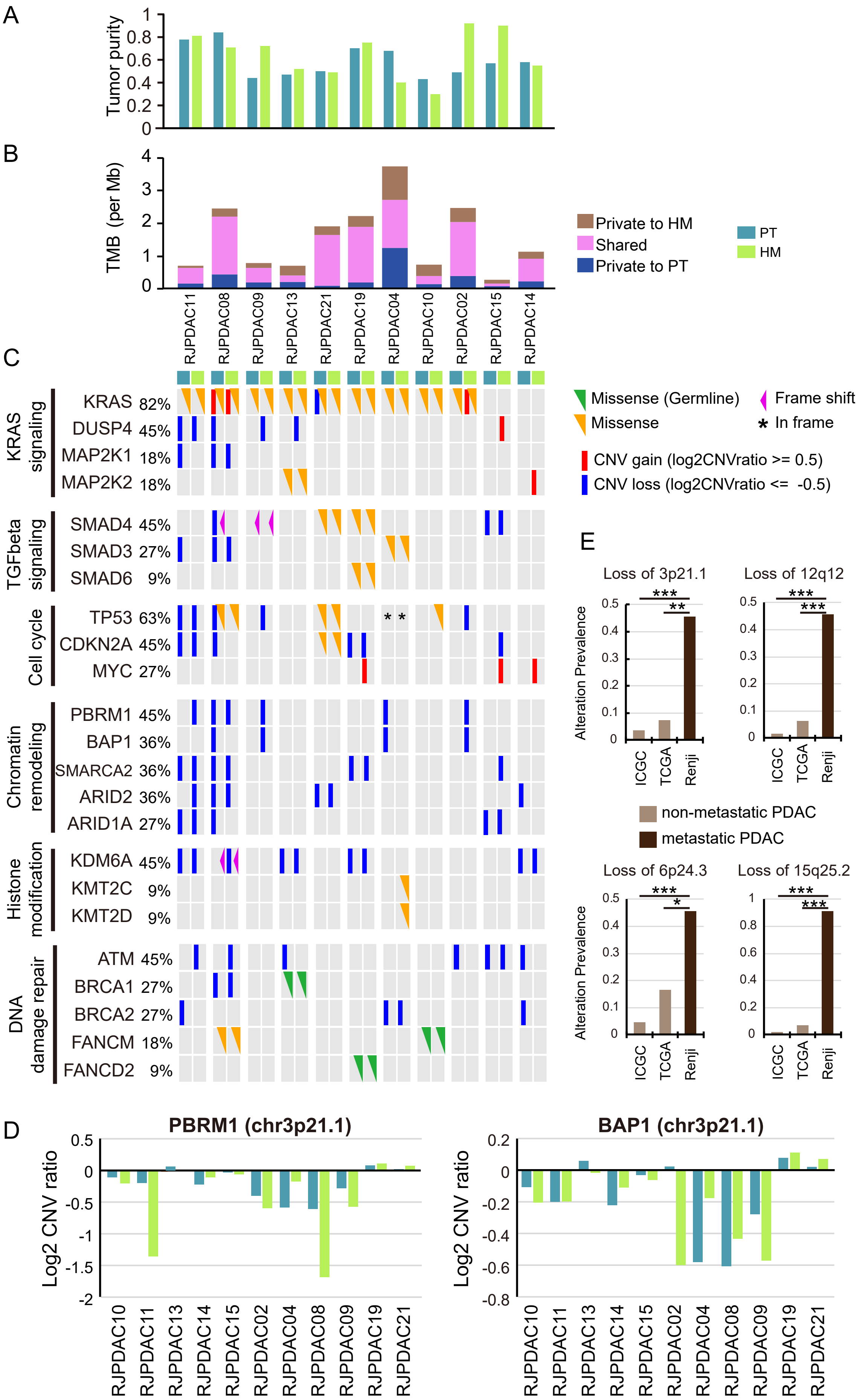

A
Tumor purity
1
0.8
0.6
0.4
0.2
0
B
TMB (per Mb)
4
3
2
1
0
Private to HM
Shared
Private to PT
PT
HM
RJPDAC11
RJPDAC08
RJPDAC09
RJPDAC13
RJPDAC21
RJPDAC19
RJPDAC04
RJPDAC10
RJPDAC02
RJPDAC15
RJPDAC14
C
KRAS signaling
KRAS 82%
DUSP4 45%
MAP2K1 18%
MAP2K2 18%
TGFbeta signaling
SMAD4 45%
SMAD3 27%
SMAD6 9%
Cell cycle
TP53 63%
CDKN2A 45%
MYC 27%
Chromatin remodeling
PBRM1 45%
BAP1 36%
SMARCA2 36%
ARID2 36%
ARID1A 27%
Histone modification
KDM6A 45%
KMT2C 9%
KMT2D 9%
DNA damage repair
ATM 45%
BRCA1 27%
BRCA2 27%
FANCM 18%
FANCD2 9%
Missense (Germline)
Missense
Frame shift
In frame
CNV gain (log2CNVratio >= 0.5)
CNV loss (log2CNVratio <= -0.5)
E
Loss of 3p21.1
Loss of 12q12
Loss of 6p24.3
Loss of 15q25.2
Alteration Prevalence
ICGC
TCGA
Renji
non-metastatic PDAC
metastatic PDAC
D
PBRM1 (chr3p21.1)
BAP1 (chr3p21.1)
Log2 CNV ratio
RJPDAC10
RJPDAC11
RJPDAC13
RJPDAC14
RJPDAC15
RJPDAC02
RJPDAC04
RJPDAC08
RJPDAC09
RJPDAC19
RJPDAC21

Fig S2

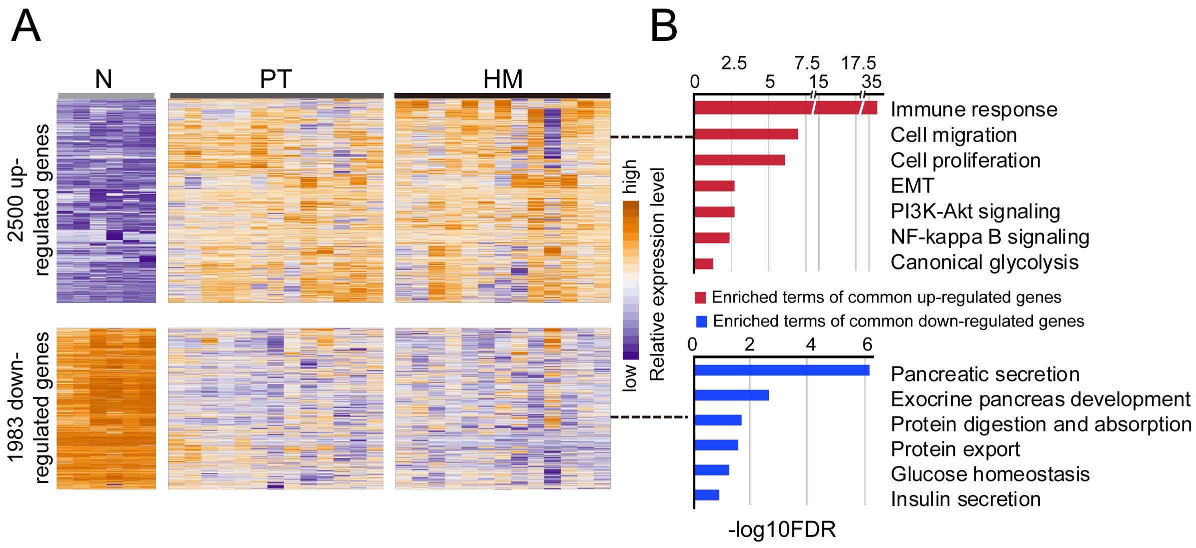

A
N
PT
HM
2500 up-regulated genes
1983 down-regulated genes
high
low
Relative expression level
B
2.5
7.5
17.5
0
5
15
35
Immune response
Cell migration
Cell proliferation
EMT
PI3K-Akt signaling
NF-kappa B signaling
Canonical glycolysis
Enriched terms of common up-regulated genes
Enriched terms of common down-regulated genes
0
2
4
6
Pancreatic secretion
Exocrine pancreas development
Protein digestion and absorption
Protein export
Glucose homeostasis
Insulin secretion
-log10FDR

Fig S3

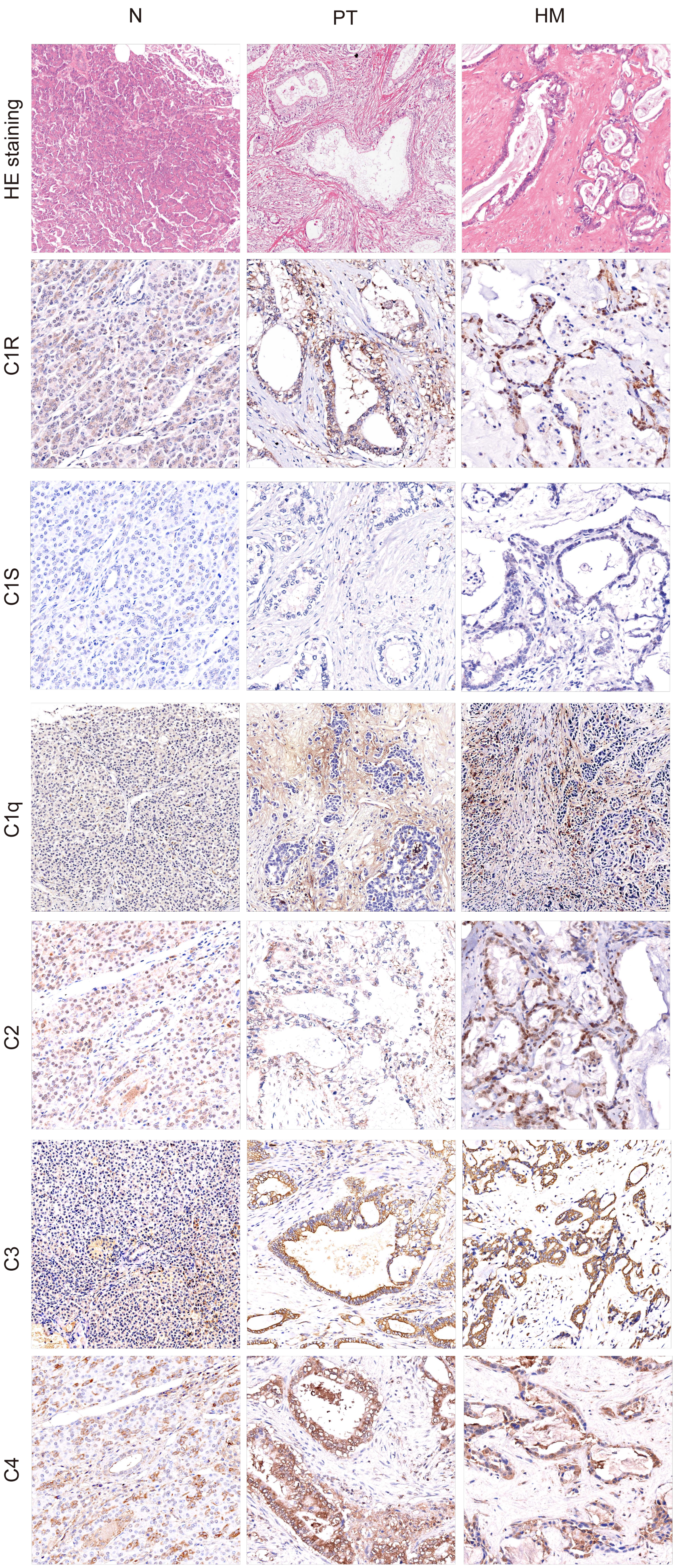

Fig S4

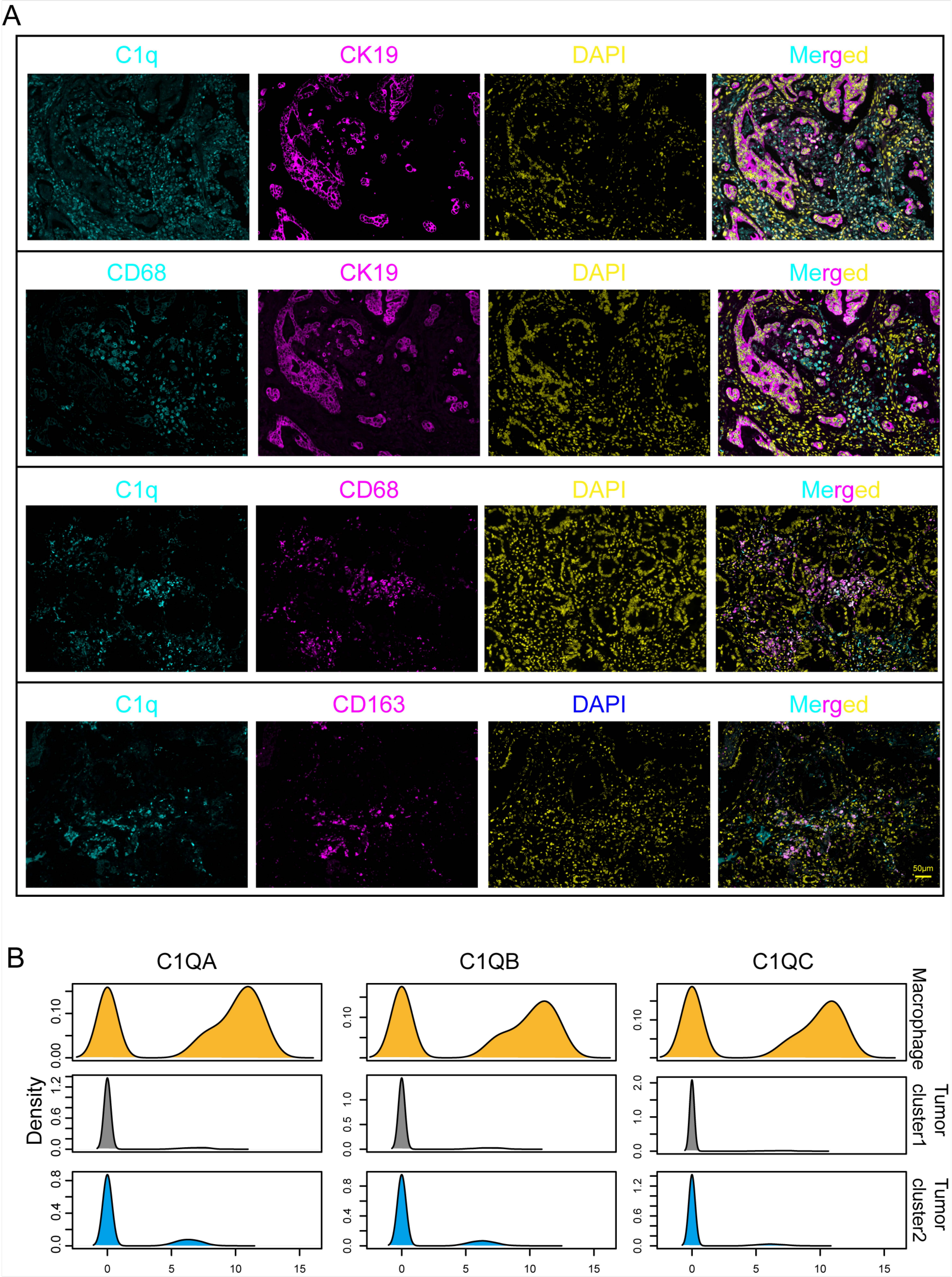

A
C1q
CK19
DAPI
Merged
CD68
CK19
DAPI
Merged
C1q
CD68
DAPI
Merged
C1q
CD163
DAPI
Merged
50μm
B
C1QA
C1QB
C1QC
Density
Macrophage
Tumor cluster1
Tumor cluster2
0
5
10
15

Fig S5

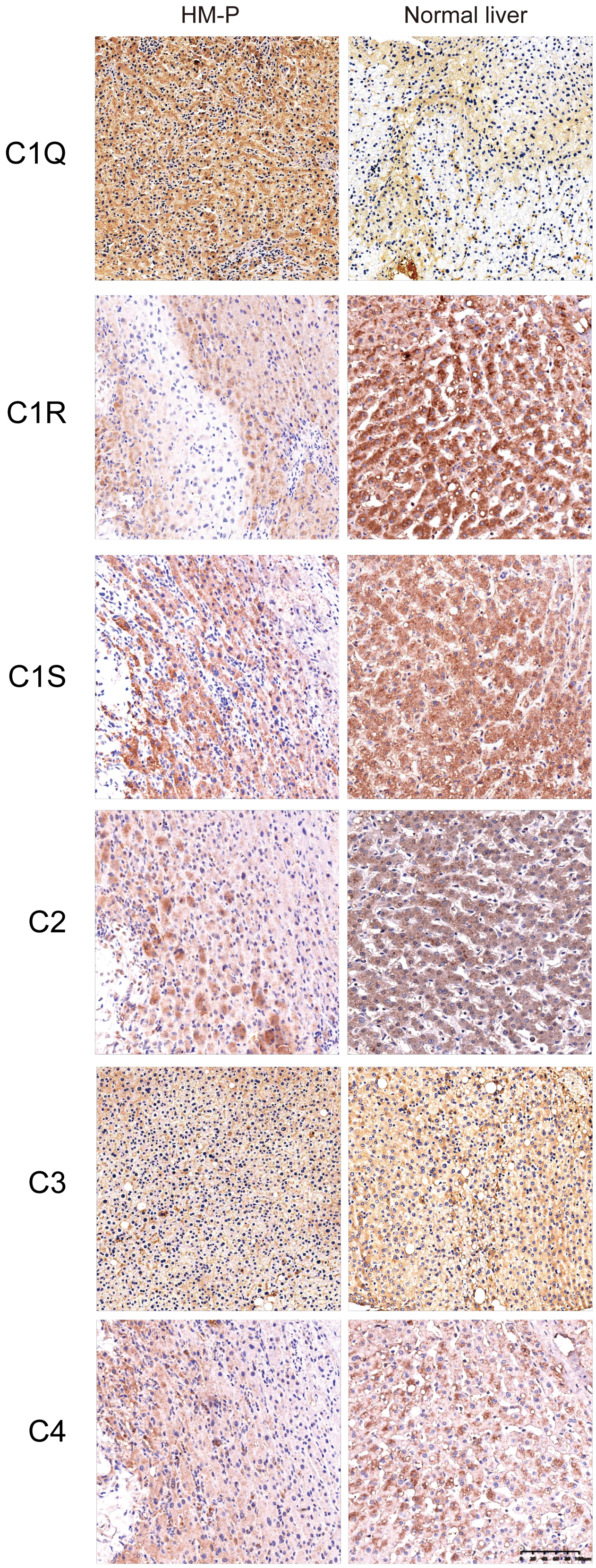

Fig S6

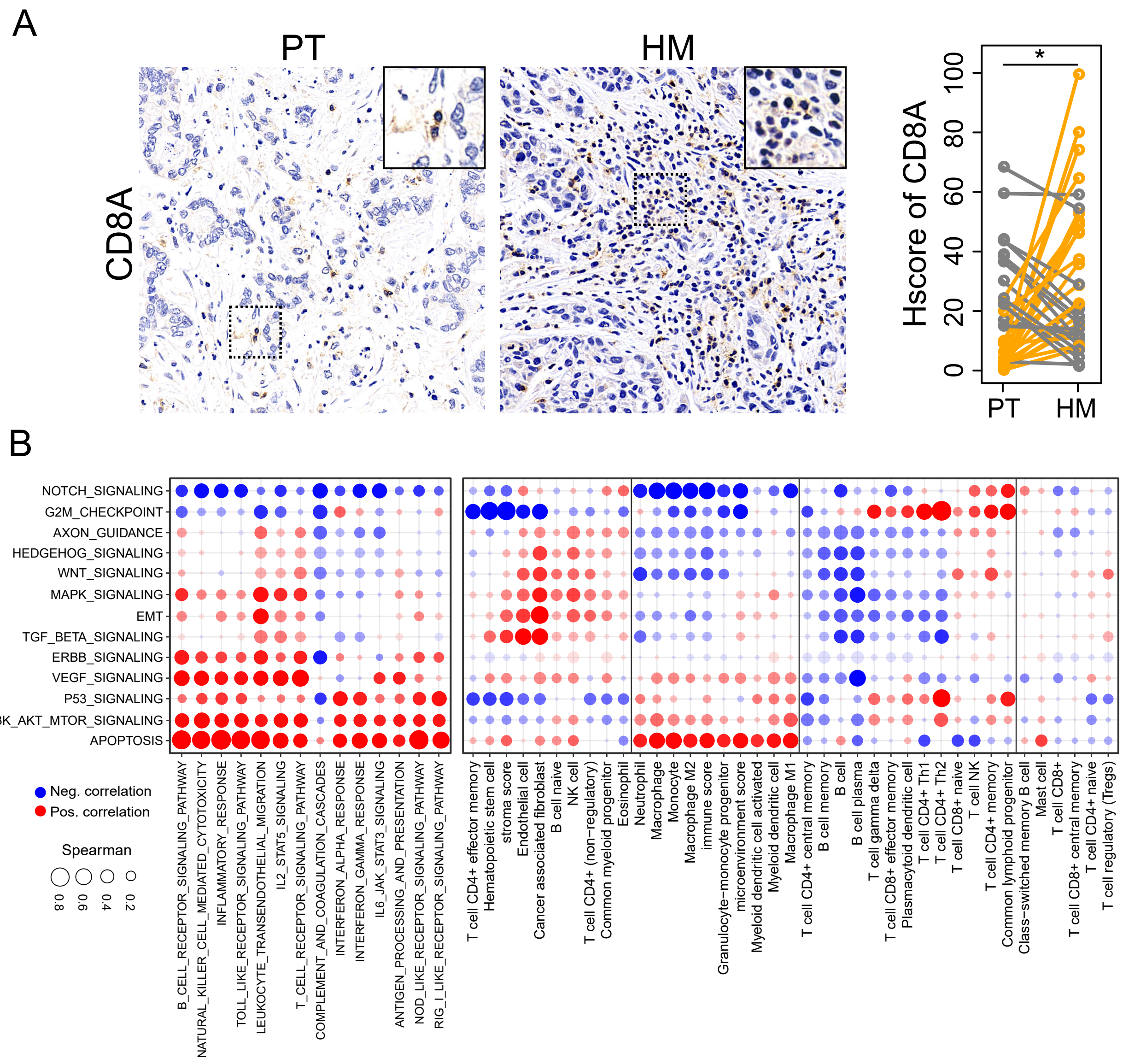
A
PT
HM
CD8A
*
Hscore of CD8A
0
20
40
60
80
100
PT
HM
B
NOTCH_SIGNALING
G2M_CHECKPOINT
AXON_GUIDANCE
HEDGEHOG_SIGNALING
WNT_SIGNALING
MAPK_SIGNALING
EMT
TGF_BETA_SIGNALING
ERBB_SIGNALING
VEGF_SIGNALING
P53_SIGNALING
PI3K_AKT_MTOR_SIGNALING
APOPTOSIS
Neg. correlation
Pos. correlation
Spearman
0.8
0.6
0.4
0.2
B_CELL_RECEPTOR_SIGNALING_PATHWAY
NATURAL_KILLER_CELL_MEDIATED_CYTOTOXICITY
INFLAMMATORY_RESPONSE
TOLL_LIKE_RECEPTOR_SIGNALING_PATHWAY
LEUKOCYTE_TRANSENDOTHELIAL_MIGRATION
IL2_STAT5_SIGNALING
T_CELL_RECEPTOR_SIGNALING_PATHWAY
COMPLEMENT_AND_COAGULATION_CASCADES
INTERFERON_ALPHA_RESPONSE
INTERFERON_GAMMA_RESPONSE
IL6_JAK_STAT3_SIGNALING
ANTIGEN_PROCESSING_AND_PRESENTATION
NOD_LIKE_RECEPTOR_SIGNALING_PATHWAY
RIG_I_LIKE_RECEPTOR_SIGNALING_PATHWAY
T cell CD4+ effector memory
Hematopoietic stem cell
stroma score
Endothelial cell
Cancer associated fibroblast
B cell naive
NK cell
T cell CD4+ (non-regulatory)
Common myeloid progenitor
Eosinophil
Neutrophil
Macrophage
Monocyte
Macrophage M2
immune score
Granulocyte-monocyte progenitor
microenvironment score
Myeloid dendritic cell activated
Myeloid dendritic cell
Macrophage M1
T cell CD4+ central memory
B cell memory
B cell
B cell plasma
T cell gamma delta
T cell CD8+ effector memory
Plasmacytoid dendritic cell
T cell CD4+ Th1
T cell CD4+ Th2
T cell CD8+ naive
T cell NK
T cell CD4+ memory
Common lymphoid progenitor
Class-switched memory B cell
Mast cell
T cell CD8+
T cell CD8+ central memory
T cell CD4+ naive
T cell regulatory (Tregs)

## Supplementary Table Notes

**Table S1. Clinical characteristics of 40 enrolled patients.** The specimens used for WES or RNA sequencing were also indicated in this table.

**Table S2. Somatic mutation list.**

**Table S3. Significantly occurred CNV regions identified by GISTIC2.** “N.S.”: not significant.

## Supplementary Figure Legend

**Fig S1. Mutation profiles of paired PTs and HMs.** A) Tumor purity of all tumor samples assessed by WES; B) Tumor mutation burden of individual samples; C) Mutation profiles of highly altered genes across samples. Genes were grouped by their function. Among them, the detailed log2ratio values of PBRM1 and BAP1 of paired samples were indicated in D); E) Highlighted CNV events which exhibited significantly increased alteration prevalence in metastatic PDACs than that in non-metastatic PDACs. *: $0.01<P<0.05$; **: $0.001<P<0.01$; ***: $P<0.001$.

**Fig S2. Expression pattern A) and enriched pathways B) of common DEGs.**

**Fig S3. IHC staining of key components of classical complementary pathways in PT and HM specimens.** C1R, C1S, C2 and C4 were mainly stained in tumor cells. C3 was expressed mainly in tumor cells of PTs. But in HM, C3 was expressed both in tumor cells and tumor stroma. Only C1q expressed in tumor stroma of both PTs and HMs. HE staining results were showed in the top panel.

**Fig S4. IF staining of C1q, macrophage cell marker and epithelial cell marker in tumor specimens as well as single-cell RNAseq data analysis of the resources of C1Q.** A) IF staining of C1q and CK19 showed that C1q was mainly expressed in tumor stroma (top panel). IF staining results of CD68 and CK19 of the same patient as in the top panel were showed in second panel. Tissue slices of top two panel are sequential slices from same patient. IF staining of C1q and CD68 showed in third panel revealed that C1q in tumor stroma was mainly derived from macrophage. IF staining of C1q and CD163 showed in last panel revealed that M2 macrophage was the main contributor of C1q. B) Peng. et al. identified macrophages and two clusters of tumor cells in primary tumor tissues of PDAC [1]. Most of tumor cells exhibited zero count in C1QA, C1QB, and C1QC while large proportion of macrophages expressed C1QA, C1QB, or C1QC (UMI>=5).

**Fig S5**. **IHC staining of key components of classical complementary pathways in HM adjacent liver tissue and normal liver tissue.** Compared to normal liver, HM adjacent normal tissue (HM-P) showed increased protein level of C1Q. To be noted, this pattern was only displayed in C1Q while C1R, C1S, C2, C3, and C4 didn't display the same pattern.

**Fig S6. Correlation of oncogenic pathways and tumor immunity.** A) HMs had increased abundance of CD8+ T cell compared to corresponding PTs. *: $p<0.05$. B) Spearman correlation between oncogenic pathways and immune-related pathways (left panel). And Spearman correlation between pathway NES values and xCell estimations of tumor infiltrating immune cells.

## Reference

1. Peng J, Sun BF, Chen CY, Zhou JY, Chen YS, Chen H, Liu L, Huang D, Jiang J, Cui GS, et al: **Single-cell RNA-seq highlights intra-tumoral heterogeneity and malignant progression in pancreatic ductal adenocarcinoma.** *Cell Res* 2019, **29:**725-738.
