# Supplementary material for: Integrated genomic and transcriptomic analysis reveals unique characteristics of hepatic metastases and pro-metastatic role of complement C1q in pancreatic ductal adenocarcinoma
Source: Genome Biol. 2021 Jan 4;22:4. doi: 10.1186/s13059-020-02222-w (PMC7780398; doi:10.1186/s13059-020-02222-w)
Supplement: Supplementary file 4 — Additional file 4. Review history. [file 13059_2020_2222_MOESM4_ESM.doc]

Review History

**First round of review**

**Reviewer 1**

**Are you able to assess all statistics in the manuscript, including the appropriateness of statistical tests used?** No, I do not feel adequately qualified to assess the statistics.

**Comments to author:**

This study by Yang et al addresses an important and understudied aspect of pancreatic cancer biology: oligometastatic liver metastases. The authors have previously performed resection and metastasectomy in a cohort of treatment naïve patients with primary pancreatic cancer and synchronous liver metastasis for which they have published their findings. They now report the DNA and RNA sequencing analyses of these matched pairs from an impressive 41 patient cohort with oligometastatic liver metastasis. The exome sequencing analyses and interpretation appear robust and the finding of 3p loss in metastases is interesting. The expression analyses are also of interest. Overall there is a high level of enthusiasm for this study that will be an important contribution to the literature. I do have some suggestions for improvement though.

1. As control populations the authors have used 105 nonmetastatic PDAs and TCGA or ICGC data. An important control that would greatly add to the study would be hepatic metastases from patients who do not have oligometastatic hepatic metastasis. These need not be matched pairs. Core biopsies would suffice, or small hepatic metastases sampled at surgery in patients whose resection have been aborted.

2. While the RNAseq data appears clean, many of the images showing validation in tissue are not convincing. As the authors know changes in RNA summarized across many samples do not necessarily translate to changes in protein expression, or the IHC is not sensitive enough to detect potentially true differences found by RNAseq. Examples of images that need to be retaken or substituted with a better example are Panel 4B that intends to illustrate lower TGFB1 in HM compared to PT, C1q images in Figure 5D and 5E. In 5D in particular there are not normal ductal structures in the image which is the true comparison to be made with the PT. The image shown in 5D is all acinar tissue. The same issue applies to the normal tissue shown in 5E. Furthermore, differences in C1q are not convincing across the spectrum of samples shown. I also suggest higher power images for these markers and arrows pointing to the regions of interest to help the reader.

3. The images in Supplemental Figure 3 are also of poor quality. Many do not appear to show the tumor at all and the staining of the hepatocytes all looks like non-specific background. H&E images of the same sections would help greatly.

4. The IF images in 5G are not convincing. An epithelial marker should be included. The cell clumps highlighted appear to be red blood cells.

5. I understand the authors meaning, but use of the terms "activated" when referring to higher expression of components of a signaling pathway are misleading. I suggest using terms such as upregulation, downregulation, etc. Activation usually implies of a functional assay.

**Reviewer 2**

**Are you able to assess all statistics in the manuscript, including the appropriateness of statistical tests used?** Yes, and I have assessed the statistics in my report.

**Comments to author:**

General comments

The manuscript by Yang and colleagues describes a study using genomic and transcriptomic analyses of primary tumors and paired synchronous hepatic metastases from patients with pancreatic cancer. Since this is the first study to report transcriptome of matched primary tumor and liver metastases it is of interest to a broader audience. The study was carried out using state-of the art experimental methods including also validation data using IHC and tissue microarrays. Several findings including 3p21.11 loss, different activations of TGFß and WNT pathways in primary tumors and liver metastases, and the contribution of the complement system are novel and might have translational implications. However, there are several issues that need to be addressed in order to improve the manuscript.

Specific comments

1. The choice of the CIBERSORT algorithm is questionable which can be also seen in the unexpected results in Figure 3D showing common immune infiltrates in primary tumors and metastases. One would expect highly divergent infiltrates due to the different organ context. Of note, the original CIBERSORT algorithm was developed based in microarray data which has different dynamic range. The authors should use the improved version CIBERSORTx (Newman et al., Nat Biotech 2019) or one of the alternative deconvolution methods (see review PMID: 31515541).

2. The activation of the different oncogenic pathways (chapter on EMT and rewiring of metabolism) in the primary tumor and the metastases should be analyzed in depth. TGFß is immunosuppressive and WNT signaling has profound impact on the immune evasion (see PMID: 29326431). Hence analyses of the association of these pathways with the immune infiltrates (and possibly IHC confirmation) could provide novel insights.

3. Albeit the number of patients used for the survival analysis in Figure 2C-E is small, the results are intriguing since the ICGC/TGCA cohorts did not show these associations. Were the ICGC/TCGA analyses done using the same algorithm (ABSOLUTE) or were these data obtained from SNP arrays?

4. Minor: the authors should use page numbers in the revised version of the manuscript.

**Point-By-Point responses:**

**Reviewer 1:**

**Comment 1:** As control populations the authors have used 105 nonmetastatic PDAs and TCGA or ICGC data. An important control that would greatly add to the study would be hepatic metastases from patients who do not have oligometastatic hepatic metastasis. These need not be matched pairs. Core biopsies would suffice, or small hepatic metastases sampled at surgery in patients whose resection have been aborted.

**Response:**

Thank you very much for your careful review of our manuscript and your kindly suggestion. The molecular features of hepatic metastases from PDAC cases with multiple metastases are also a big interest of us. And we are wishing to explore such samples. Unfortunately, the acquirement of such sample is quite difficult. Basing on precise preoperative assessment by high resolution enhanced abdominal computed tomography (CT) and/or magnetic resonance imaging (MRI), there is few accidental PDAC patients developing extensive metastases in current clinical practice. Additionally, PDAC is characterized by abundant tumor stoma leading to low success rate of core biopsy to collect tumor cells. There are often insufficient biopsy specimens for IHC and sequencing after histopathological examination. Moreover, such patients are considered unfit for surgery. Therefore, there are currently not insufficient specimens for us to conduct further analysis.

However, we managed to search for publications that assessed the molecular profiles of metastases collected from autopsy specimens from PDAC cases with multi-metastases (PMID: 20981102 and PMID: 28092682). The details are showed in Table R1 in this response letter. In brief, KRAS, TP53, CDKN2A, and SMAD4 were frequently altered in PDAC cases with oligometastases or multi-metastases. And chr3p21.1 is also frequently deleted in PDAC cases with multiple metastases. And we revised our manuscript at line 143-145 in page 6.

**Table R1. Highly altered genomic regions in PDAC metastases.**

Note: Loss of 3p21.1 in (PMID: 28092682) was assessed as copy number deletion of PBRM1.

“NA”: Not available.

| **­** | Our manuscript | 20981102 | 28092682 |
| --- | --- | --- | --- |
| **Num. Patient** | 40 (11 with WES data) | 7 | 4 |
| **Tissue** | Paired primary tumors and oligometastases | Low passage cell line or first passage PDXs from metastatic lesions (3 liver, 2 lung, and 2 Peritoneum) | 26 metastases |
| **Sample Type** | Fresh samples | Autopsy | Autopsy |
| **Highly altered genes** | KRAS, CDKN2A, TP53, SMAD4, PBRM1, KDM6A, ATM | KRAS (clonal) | KRAS, CDKN2A, TP53, SMAD4, ARID1A, ATM (all clonal) |
| **3p21.1 loss** | 5 (45.5%) | 6 LOH (85.7%) | 4 (100%) |
| **6p24.3 loss** | 5 (45.5%) | 3 LOH (42.86%) | NA |
| **8q23.1 gain** | 10 (90.9%) | 4 CNV gain (1 exhibit very high copy) | NA |
| **12q12 loss** | 5 (45.5%) | 3 (42.86%) | NA |
| **13q12.13 loss** | 10 (90.9%) | 0 | NA |
| **15q25.2 loss** | 10 (90.9%) | 3 (all whole chromosome LOH, 42.86%) | NA |
| **16p13.3 gain** | 6 (54.5%) | 5 (4 whole chromosome gain, 71.4%) | NA |

**Comment 2:** While the RNAseq data appears clean, many of the images showing validation in tissue are not convincing. As the authors know changes in RNA summarized across many samples do not necessarily translate to changes in protein expression, or the IHC is not sensitive enough to detect potentially true differences found by RNAseq. Examples of images that need to be retaken or substituted with a better example are Panel 4B that intends to illustrate lower TGFB1 in HM compared to PT, C1q images in Figure 5D and 5E. In 5D in particular there are not normal ductal structures in the image which is the true comparison to be made with the PT. The image shown in 5D is all acinar tissue. The same issue applies to the normal tissue shown in 5E. Furthermore, differences in C1q are not convincing across the spectrum of samples shown. I also suggest higher power images for these markers and arrows pointing to the regions of interest to help the reader.

**Response:**

Thank you very much for your careful review and kindly suggestions. We have provided images in higher power to substitute the original images (Figure 4B, Figure 5D, Figure 5E). And images of tumor-adjacent normal samples that containing ductal structure were provided. In addition, we pointed out the regions of interest and magnified it on the top right corner of the picture in Figure 4 and Figure 5 to help the reader.

**Comment 3:** The images in Supplemental Figure 3 are also of poor quality. Many do not appear to show the tumor at all and the staining of the hepatocytes all looks like non-specific background. H&E images of the same sections would help greatly.

**Response:**

Thank you very much for your careful review of our manuscript. We are very sorry for the confusion in understanding. We checked the images in Supplementary Figure 3 and consults experienced pathologists to make sure that we provided images of tumor lesions. We selected typical tumor staining regions in PT and HM. Therefore, there were not hepatocytes in the images of HM. We noted that different classical complements would exhibit different levels and locations of staining. All complement proteins except C1q was stained in same pathological section, so we made the H&E images in the same sections to make easy for readers to understand.

­

**Comment 4:** The IF images in 5G are not convincing. An epithelial marker should be included. The cell clumps highlighted appear to be red blood cells.

**Response:**

Thank you very much for your careful review of our manuscript. We are very sorry for the confusion in understanding. In order to show the location and origin of C1q more clearly, we performed the immunofluorescence (IF) staining for an epithelial marker (CK19)/C1q, CK19/CD68, CD68/C1q, CD163/C1q (Supplementary Figure 4A and Figure R1 in this response letter). IF staining of C1q and CK19 showed that C1q was mainly expressed in tumor stroma (top panel of Supplementary Figure 4A). IF staining results of CD68 and CK19 from the sequential slice of the same patient showed in top panel were provided in second panel. IF staining of C1q and CD68 showed in third panel revealed that C1q in tumor stroma was mainly derived from macrophage. IF staining of C1q and CD163 showed in last panel revealed that M2 macrophage was the main contributor of C1q. And we replaced the original images in Figure 5G with those of higher power.

Figure R1. Immunofluorescence assays demonstrated C1q was co-localized with macrophage.

**Comment 5**: I understand the authors meaning, but use of the terms "activated" when referring to higher expression of components of a signaling pathway are misleading. I suggest using terms such as upregulation, downregulation, etc. Activation usually implies of a functional assay.

**Response:**

We have revised our manuscript and replaced the terms “activated” with terms such as “upregulated”, “upregulation”, “downregulation”, or “downregulated” at line 199-202 in page 8, 217-219 in page 8, 223-225 in page 8, and 311-313 in Page 12.

**Reviewer 2:**

**Comment 1:** The choice of the CIBERSORT algorithm is questionable which can be also seen in the unexpected results in Figure 3D showing common immune infiltrates in primary tumors and metastases. One would expect highly divergent infiltrates due to the different organ context. Of note, the original CIBERSORT algorithm was developed based in microarray data which has different dynamic range. The authors should use the improved version CIBERSORTx (Newman et al., Nat Biotech 2019) or one of the alternative deconvolution methods (see review PMID: 31515541).

**Response:**

Thank you very much for your careful review of our manuscript. The methods and the review (PMID: 31515541) you recommended offered great help for us. We employed both CIBERSORTx and xCell algorithms to estimate the abundance of tumor infiltrating immune cells. For a specific sample, we identified top abundant types of immune cell within TME by using CIBERSORTx in “relative” mode which allows between-cell type comparisons (PMID: 31515541). As xCell is capable of deciphering dozens of types of immune cells and the result of which is allowed for between-sample comparison (PMID: 31515541), we identified differentially infiltrated immune cells between paired PTs and HMs using the results of xCell. The xCell algorithm that packed in immundeconv R package is used. TPM (transcript per million) matrix is used as input for both CIBERSORTx and xCell. The corresponding methods were revised on “Method” section (line 545-554 at page 19 of revised paper). Both xCell and CIBERSORTx (absolute mode) suggested the increase of abundance of M2 macrophage in HM compared to PTs (See Figure R2 in this response letter).

Figure R2. Estimation of Tumor infiltrating M2 macrophage by CIBERSORTx (absolute mode, left) and xCell (right). *: p< 0.05, **: p<0.01.

**Comment 2:** The activation of the different oncogenic pathways (chapter on EMT and rewiring of metabolism) in the primary tumor and the metastases should be analyzed in depth. TGFß is immunosuppressive and WNT signaling has profound impact on the immune evasion (see PMID: 29326431). Hence analyses of the association of these pathways with the immune infiltrates (and possibly IHC confirmation) could provide novel insights.

**Response:**

Thank you very much for your careful review of our manuscript. And thank you very much for your kindly suggestions. Indeed, the analyses of the association of oncogenic pathways with the immune infiltrates do provide new insights. As showed in the Figure R3 in this response letter, we firstly experimentally observed the increase of abundance of CD8+ T cell in HMs compared to corresponding PTs. *: p<0.05 (Figure R3A). Then we calculated Spearman correlation of GSVA NES scores between oncogenic pathways and immune-related pathways and immune cell infiltration (Figure R3B). Surprisingly, NOTCH signaling exhibited more significantly negative correlated with immune infiltrates, while apoptosis exhibited most significantly positive correlation with immune infiltrates. When looking into the detail expression change of NOTCH signaling across individuals, we found ligands (DLL4, DLL1, JAG1, JAG2), receptors (NOTCH1, NOTCH3, and NOTCH4), and downstream targets (HES1 and HES5) exhibited consistently decreased expression in HMs compared to PTs (Figure R3C). Together, these suggested the combination of NOTCH inhibitor and immune therapy might achieve better clinical efficacy for oligometastatic PDAC patients. Thus, we plan to set up a new project to study the association of NOTCH signaling with PDAC metastasis and its clinical potential. Once again, thank you, thank you very much for your suggestions!

The corresponding descriptions in revised manuscript were showed at line 348 to 354 in page 13 and line 557 and 563 in page 19.

Figure R3. Association of oncogenic pathway and tumor immunity

**Comment 3**: Albeit the number of patients used for the survival analysis in Figure 2C-E is small, the results are intriguing since the ICGC/TGCA cohorts did not show these associations. Were the ICGC/TCGA analyses done using the same algorithm (ABSOLUTE) or were these data obtained from SNP arrays?

**Response:**

Thank you very much for your careful review of our manuscript. We apologize for the unclear description of method details. Since we have no access to the raw data deposited in TCGA and ICGC, we could only retrieve the public level-3 data(segment files of copy number). The copy number data from both TCGA and ICGC were generated from SNP arrays. TCGA employed DNAcopy R package to estimate copy number alterations. ICGC SNP arrays were scanned and data was processed using the Genotyping module (v1.8.4) in Genomestudio v2010.3 (Illumina, San Diego CA) to calculate B-allele frequencies (BAF) and logR values. Items in both segment files were masked for germline variations.

In our analysis, the same cutoff of |segment_mean| was used for all these data. Genomic regions with segment mean smaller than -0.3 are categorized as copy number loss while those with segment mean greater than 0.3 are categorized as copy number gain. The corresponding method details are revised (line 501-505 at page 18).

**Comment 4:** Minor: the authors should use page numbers in the revised version of the manuscript.

**Response:**

Thanks for the reviewer’s kindly suggestions. The page numbers were given at the footer.

**Second round of review**

**Reviewer 1**

My concerns have been addressed. The histologic images are of much greater quality and greatly add to the story.

**Reviewer 2**

he authors addressed all issues raised in the original review satisfactorily and thereby improved the manuscript. Specifically, they carried out additional analyses using the CIBERSORTx and xCell methods and confirmed the increase of M2 macrophages in metastatic compared to primary tissues. Additionally, as suggested, the authors investigated the association between the immune infiltrates and oncogenic pathways and identified NOTCH signalling. Finally, the question regarding the SNP arrays has been addressed

Minor fix on page 4, line 103: age numbers should not be provided with three digits, use 62.2 instead.
